# Decrease in coccolithophore calcification and CO_2_ since the middle Miocene

**DOI:** 10.1038/ncomms10284

**Published:** 2016-01-14

**Authors:** Clara T. Bolton, María T. Hernández-Sánchez, Miguel-Ángel Fuertes, Saúl González-Lemos, Lorena Abrevaya, Ana Mendez-Vicente, José-Abel Flores, Ian Probert, Liviu Giosan, Joel Johnson, Heather M. Stoll

**Affiliations:** 1 Geology Department, Oviedo University, Arias de Velasco s/n, 33005 Oviedo, Asturias, Spain; 2 Aix-Marseille University, CNRS, IRD, CEREGE UM34, 13545 Aix en Provence, France; 3 Grupo de Geociencias Oceánicas, Geology Department, University of Salamanca, Salamanca 37008, Spain; 4 CNRS, Sorbonne Universités-Université Pierre et Marie Curie (UPMC) Paris 06, FR2424, Roscoff Culture Collection, Station Biologique de Roscoff, Place Georges Teissier, 29680 Roscoff, France; 5 Department of Geology and Geophysics, Woods Hole Oceanographic Institution, 266 Woods Hole Road, MS# 22, Woods Hole, Massachusetts 02543-1050, USA; 6 University of New Hampshire, Department of Earth Sciences, 56 College Road, James Hall, Durham, New Hampshire 03824-3589, USA

## Abstract

Marine algae are instrumental in carbon cycling and atmospheric carbon dioxide (CO_2_) regulation. One group, coccolithophores, uses carbon to photosynthesize and to calcify, covering their cells with chalk platelets (coccoliths). How ocean acidification influences coccolithophore calcification is strongly debated, and the effects of carbonate chemistry changes in the geological past are poorly understood. This paper relates degree of coccolith calcification to cellular calcification, and presents the first records of size-normalized coccolith thickness spanning the last 14 Myr from tropical oceans. Degree of calcification was highest in the low-pH, high-CO_2_ Miocene ocean, but decreased significantly between 6 and 4 Myr ago. Based on this and concurrent trends in a new alkenone *ɛ*_p_ record, we propose that decreasing CO_2_ partly drove the observed trend via reduced cellular bicarbonate allocation to calcification. This trend reversed in the late Pleistocene despite low CO_2_, suggesting an additional regulator of calcification such as alkalinity.

## Abstract (web summary)

The impact of future and past carbonate chemistry changes on calcifying plankton is poorly understood. Here, the authors show that coccolithophore degree of calcification decreased significantly between 6 and 4 million years ago, in line with declining aqueous CO_2_ concentrations.

## Graphical abstract

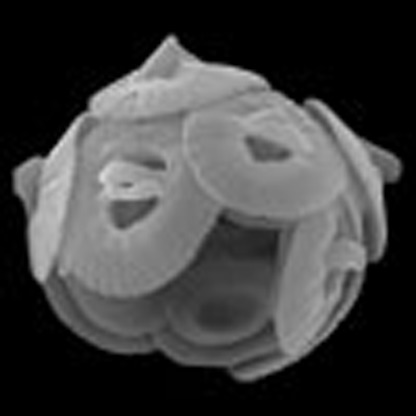

Coccolithophores, a group of unicellular marine phytoplankton, are the only primary producers of biogenic calcite in the open ocean. During their diploid life-cycle stage, calcifying coccolithophores intracellularly produce calcite plates called heterococcoliths. These circular to elliptical coccoliths are extruded through the cell wall to form an exoskeleton, usually composed of a single layer of calcite plates, called a coccosphere. Coccolithophores play an important role in the carbon cycle because they promote the sinking of particulate organic carbon to the deep ocean^1^. Changes in their production of organic carbon and calcification can alter the balance between the organic and inorganic carbon pumps, with strong feedbacks on climate and atmospheric carbon dioxide concentrations (*p*CO_2_) on seasonal to geological timescales^2^. Despite the importance of coccolithophore calcification to biogeochemical cycles and the large range in degree of cell-calcification (defined here as the amount of calcite per unit surface area of the cell) observed both among and within modern species, it is unclear whether specific factors drive changes in cell-calcification state of the ocean's coccolithophore populations on evolutionary timescales. Rapid changes in ocean dissolved CO_2_ concentration ([CO_2aq_]), pH, temperature and surface-water stratification in the coming centuries may exert selective pressure on coccolithophore calcification^3^,^4^. Short-term experiments reveal an array of species- and strain-specific physiological responses to elevated [CO_2aq_]^4^,^5^,^6^,^7^,^8^,^9^. However, selection experiments lasting around a year show that the negative effects of short term (<10 generations) high *p*CO_2_ exposure on coccolithophore calcification and growth are partly reversed for populations that have been exposed to long term (about 500 generations) high *p*CO_2_ conditions^10^,^11^. Such adaptability is consistent with the geological data indicating that coccolithophores were more ubiquitous and common in the warm, high-CO_2_ ocean of the earlier Cenozoic, with larger coccoliths and cells^12^,^13^. Recent work has shown that calcification competes with photosynthesis for intracellular bicarbonate (HCO_3_^−^), and that multiple species of coccolithophores reallocate HCO_3_^−^ transport from calcification to photosynthesis at low [CO_2aq_]^14^.

Here we explore the long-term response of coccolithophore calcification and HCO_3_^−^ allocation to the evolution of ocean conditions and [CO_2aq_] over the past 14 million years (Myr) in a key coccolithophore family, the Noëlaerhabdaceae. This family, which includes the genera *Emiliania*, *Gephyrocapsa, Pseudoemiliania* and *Reticulofenestra*, dominates most modern ocean coccolithophore communities as well as fossil assemblages since the Miocene. As an indicator of coccolithophore calcification, we show that coccolith thickness correlates strongly with cellular calcification per unit surface area across the range of modern Noëlaerhabdaceae. We then document changes in the size and degree of calcification of Noëlaerhabdaceae coccoliths since 14 Myr ago in two sediment sequences from the tropical Atlantic and Indian Oceans containing well-preserved coccoliths (Ocean Drilling Program, ODP, Site 925 and Indian National Gas Hydrate Program Site NGHP-01-01A, respectively; Fig. 1). In contrast to recent studies of coccolith mass^5^,^15^,^16^,^17^,^18^, we present our data as coccolith thickness within narrow size classes to focus on changes in degree of calcification of coccoliths, allowing us to better capture the potential coccolithophore calcification response to changes in the palaeo-carbonate system^19^. These records are the first to document past long-term changes in coccolithophore calcification for given cell size classes in the Miocene and Pliocene, when *p*CO_2_ was higher than pre-industrial levels. Using the geochemical signature of coccoliths, we then assess if changes in degree of calcification correspond to changes in the allocation of intracellular HCO_3_^−^ resources to calcification. Finally, we evaluate the potential role of changing upper ocean stratification and [CO_2aq_] on both degree of calcification and HCO_3_^−^ resource allocation, using new proxy records of foraminiferal stable isotopes (*δ*^18^O and *δ*^13^C) and carbon isotopic fractionation by alkenone-producing haptophyte algae during photosynthesis (*ɛ*_p_).

## Results

### Coccolith thickness and cellular calcification

New culture experiments sampling the diversity of modern Noëlaerhabdaceae coccolithophores show variation in coccolith thickness both among the different species and among different strains of the same species. This variation in thickness correlates strongly with variation in cellular calcification per surface area as well as with changes in calcite/organic carbon, a measure of calcification per cell volume (Fig. 2 and Supplementary Fig. 1). The thickness of an individual coccolith is intimately linked to the degree of calcification of a cell, because it represents a key mechanism by which cells can regulate the amount of biomineral for a given cell volume. Various factors may drive cells to adjust calcite per cell surface area. In this study, we focus on changes that are occurring within narrow size classes. In addition, calcite per cell surface area varies with cell size across the modern diversity of placolith-bearing coccolithophores, where small cells are characterized by thinner coccoliths (Supplementary Fig. 2). This latter effect may be an adaptation to compensate for the higher surface area to volume ratio of small cells that, if calcification per cell surface area were constant across all cell sizes, would impose a much higher biomineral requirement relative to cell volume in small cells. While coccolith mass has been used as an indicator of cellular calcification in Pleistocene and recent sediments^15^,^16^,^17^,^18^, coccolith mass is driven by changes in cell size as well as degree of calcification. On the other hand, coccolith thickness within narrow size classes, or size-normalized coccolith thickness, represent degree of calcification and are indicators better suited to reconstructing coccolithophore calcification on long timescales over which significant coccolith and cell size changes occur. The range of coccolith thickness variation among cultured Noëlaerhabdaceae strains (Fig. 2) is consistent with previous observations that phenotypic differences in the degree of calcification between species and between strains of the same species tend to be much larger than the phenotypic plasticity of a single strain cultured under varying environmental conditions^8^,^9^,^20^. This may arise if coccolith morphotype or thickness is genetically regulated^9^. The potential for large intraspecific diversity may reflect the genetic architecture, in that the dominant modern Noëlaerhabdaceae *Emiliania huxleyi* has a pan-genome composed of core genes plus genes distributed variably amongst strains^21^.

### Decreasing cellular calcification since the late Miocene

Over the past 14 Myr, the Noëlaerhabdaceae have undergone large variations in coccolith size (Fig. 3) and degree of calcification, represented by thickness (Fig. 4). Changes in coccolith thickness are evident in both narrowly restricted size classes, as well as in measurements of size-normalized (SN) thickness and calculated ‘shape factor', confirming that they are not a direct result of temporal changes in coccolith and cell size (isometric scaling, that is, changes related to proportional changes in size) (Fig. 4). The quantification of thickness was not biased by variable coccolith fragmentation (Supplementary Fig. 3). Scanning electron microscope (SEM) observations confirm that in all samples the original crystal structure of the coccolith remains well defined. Only on some older coccoliths did we identify a small amount of diagenetic overgrowth (small abiogenic crystals formed on the surface of the collar in the central area; Supplementary Data 1) However, the presence of this minor overgrowth does not correspond to an increase in coccolith thickness, except in the oldest 14 Myr old sample at the Indian Ocean Site. Thus, with this exception, the preservation visible under SEM makes it unlikely that middle Miocene Noëlaerhabdaceae coccoliths of a given size were originally thinner and more delicate than those present in our samples. This suggests that either (1) overgrowth was minor enough not to significantly impact mean coccolith thickness, or (2) the calcite that recrystallized on the surface of coccoliths was originally derived from dissolution of primary calcite of these same coccoliths. Between 8 and 3 Myr ago at both sites, *Spenolithus* and *Discoaster* nannoliths are abundant. These are typically more susceptible than placolith coccoliths to overgrowth due to their crystal structure, yet SEM images show that these susceptible forms exhibit excellent and constant preservation, providing supporting evidence that diagenetic overgrowth was not more significant when Noëlaerhabdaceae coccoliths showed a higher degree of calcification at 6–8 Myr ago relative to at 3–4 Myr ago (Supplementary Data 1; Supplementary Figs 4 and 5).

The measured coccolith populations exhibit large variability in the morphology and degree of calcification of small coccoliths within and between each sample (Fig. 4; Supplementary Figs 4 and 5; Supplementary Data 1). For example, *Gephyrocapsa protohuxleyi*, a form close to *E. huxleyi* but with a central area bridge characteristic of *Gephyrocapsa*, was present in Pleistocene samples at both sites alongside much more heavily calcified *Gephyrocapsa* coccoliths. Despite this large diversity in morphology and thickness, there are significant changes in the dominance of more heavily calcified versus more lightly calcified forms over time, as well as the emergence during the early Pliocene of coccoliths thinner and/or with larger central area openings than those found in previous intervals. Coccolith degree of calcification was on average highest between 14 and 6 Myr ago and decreased abruptly in the late Miocene to early Pliocene (6–4 Myr ago) to low values that were maintained during the Pliocene and early Pleistocene (4–1 Myr ago). For the few sample points of the last 1 Myr ago, degree of calcification increased both in the Indian and Atlantic Ocean records relative to this Pliocene minimum (Fig. 4). However, we note that assemblages in our samples <1 Myr are dominated by *Gephyrocapsa* coccoliths and pre-date the emergence of the less heavily calcified *E. huxleyi* (see *k*_*s*_ values, Fig. 4d,h), which is significant especially in modern high and mid-latitude regions. Large changes in degree of coccolith calcification, including the decrease from 6 to 4 Myr ago and the increase around 1 Myr ago at both sites, occurred within the dominant genus at a given time, and do not coincide with major shifts in the contribution of different genera to the Noëlaerhabdaceae (Fig. 4).

### Late Miocene changes in cellular HCO_3_^−^ allocation

Geochemical records of carbon isotopic fractionation into coccolith calcite (*ɛ*_coccolith_) can be used to elucidate the relationship between the observed changes in degree of calcification and the resource allocation of carbon to calcification. Models of cellular carbon fluxes have shown that *ɛ*_coccolith_ becomes increasingly depleted if the rate of supply of HCO_3_^−^ to the site of calcification (coccolith vesicle) is reduced relative to calcification rate^14^. Our new records of *ɛ*_coccolith_ from ODP Site 925 show that large cells begin to decrease the HCO_3_^−^ allocation to calcification at about 8 Myr ago, evidenced by decreasing *ɛ*_coccolith_ (Fig. 5a,b). This trend occurs shortly after a decrease in mean Noëlaerhabdaceae coccolith size (interpreted as a reduction in mean cell size^13^) at both sites (Fig. 3) that is also observed in other low-latitude records^12^,^22^,^23^. Reduced HCO_3_^−^ allocation to calcification continues in large cells from 6 to 4 Myr ago, as indicated by decreasing *ɛ*_coccolith_ during this interval, despite a stable trend in mean coccolith size. Although we cannot resolve changes in the degree of calcification of large coccoliths in this study (see Methods), the *ɛ*_coccolith_ trend suggests that in large cells, the change in HCO_3_^−^ allocation to calcification was of greater magnitude than any concurrent decrease in calcification that may have occurred. This significant reduced allocation to calcification in large cells drove a divergence in the range of vital effects among small and large coccoliths after 8 Myr ago (Fig. 5a), similar to the results from Caribbean ODP Site 999 (ref. 14).

Small coccoliths show evidence for decreased HCO_3_^−^ allocation to calcification only since 6 Myr ago. From 11 to 6 Myr ago, *ɛ*_coccolith_ and SN coccolith thickness are relatively stable (Fig. 5b,c), suggesting minimal changes in HCO_3_^−^ allocation to calcification. In contrast, between 6 and 1 Myr ago, a near-constant *ɛ*_coccolith_ indicates a stable ratio of HCO_3_^−^ allocation to the coccolith vesicle relative to calcification rate, despite a large decrease in degree of calcification (Figs 4 and 5c). This implies a decrease in HCO_3_^−^ allocation to calcification of comparable magnitude to the decrease in cellular calcification. In the last 1 Myr, an increase in degree of calcification in the small coccoliths with no change in *ɛ*_coccolith_ suggests that allocation of HCO_3_^−^ to calcification also increased in parallel.

### Relationship between calcification and ocean stratification

Water column stratification influences productivity and production depth in the tropics. Stratification can be inferred from foraminiferal *δ*^18^O gradients between the upper mixed layer (*Globigerinoides sacculifer*) and thermocline (*Globorotalia menardii*), because these reflect the upper photic zone temperature and salinity gradients^24^. The temporal evolution of planktic foraminiferal *δ*^18^O at Sites ODP 925 and NGHP-01-01A is shown in Fig. 6. Between 3.5 and 2 Myr ago, a deep thermocline at Site 925 is inferred from independent foraminiferal assemblage indicators^25^, potentially suggesting a deeper coccolithophore depth habitat and lower light levels. Decreased light has been shown to reduce cellular calcification (PIC/SA) twofold by a reduction in photon flux density from 80 to 15 μmol m^−2^ s^−1^ in culture^26^, and low light levels have been proposed to decrease cellular HCO_3_^−^ transport^27^. However, neither site shows a clear decrease in *δ*^18^O gradients at this time (Fig. 6a,b), as would be expected if reduced coccolith calcification from 4 to 1 Myr ago were due to a deepening of the thermocline, resulting in a reduced temperature gradient between the two foraminifer species' depth habitats. Proxy records suggest high productivity from 10 to 8 Myr ago in the Indian Ocean^28^ and from 6.6 to 6 Myr ago at ODP Site 925 (ref. 29). Thus, reconstructed changes in water column structure and paleoproductivity do not consistently co-vary with changes in degree of coccolith calcification.

### Calcification and [CO_2aq_] in the Miocene–Pliocene

Carbon isotopic fractionation in phytoplankton during photosynthesis (*ɛ*_p_) varies directly with [CO_2aq_] and has been widely applied as a CO_2_ proxy in the Cenozoic. However, limited data exist for the interval of major changes in calcification and HCO_3_^−^ allocation between 14 and 5 Myr ago. In addition, the interpretation of any data is complex because of the expected influence of active HCO_3_^−^ allocation on *ɛ*_p_ (ref. 14). Our new record of *ɛ*_p_ extends the published record from ODP Site 999 for the last 5 Myr^30^ back to 16 Myr ago (Fig. 7a). This extended record reveals a decrease in *ɛ*_p_ from 16 to 8 Myr ago, an excursion to higher *ɛ*_p_ values at 7 Myr ago, and then a continued decrease towards the present. The decline in *ɛ*_p_ could be driven by decreasing [CO_2aq_], increasing cellular growth rates that increase carbon demand relative to supply, or increasing cell sizes that reduce surface area to volume and thus diffusive supply (see ref. 31 and references therein). Following previous workers, [CO_2aq_] is estimated with the formula [CO_2aq_]=*b*/(*ɛ*_f_−*ɛ*_p_), where *ɛ*_f_ is a constant reflecting the maximum effective photosynthetic fractionation by the cell (25‰), and ‘*b*' encompasses factors such as growth rate and cell geometry that modulate the ratio of carbon supply to demand by the cell. First, to estimate temporal variations in *b* due to cell size, we use previous formulations of the relationship between cell size and *b*^32^, together with our record of tropical Noëlaerhabdaceae coccolith size evolution (Fig. 7b), which shows trends similar to those at other tropical sites^22^,^23^. The decrease in cell size after 9 Myr ago, compared with the average between 11 and 16 Myr ago, corresponds to a 25% reduction in the *b* value. Second, we estimate the influence of productivity on *b* using proxy records from ODP Site 999 of coccolith Sr/Ca and alkenone mass accumulation rates (Fig. 7c). These records confirm that there is no long-term productivity increase, and suggest maxima from 13 to 10 Myr ago and at 8 Myr ago. Calculated *b* values are shown in Fig. 7d. The resulting estimates of [CO_2aq_] (Fig. 7e) show a trend of continued decline over the past 16 Myr, with the exception of a local maximum at 9.3–10.3 Myr ago resulting from the unusually large cell sizes in the geometry correction. Assuming equilibrium with the atmosphere, these results are similar in trend and magnitude to [CO_2aq_] predicted from the atmospheric *p*CO_2_ curve of ref. 33 derived from inverse modelling of climate data (Fig. 7e). The absolute values of [CO_2aq_] are subject to greater uncertainty than the trend.

As in previous studies^30^, our calculations would not account for the likely increase in active carbon uptake for photosynthesis as [CO_2aq_] declined^14^,^34^, especially after 8 Myr ago. Because active carbon transport increases the chloroplast uptake of inorganic carbon relative to fixation, it can result in higher *ɛ*_p_ values than would be predicted from passive diffusive CO_2_ uptake alone^35^. Laboratory culture experiments suggest that active HCO_3_^−^ transport to the chloroplast becomes more significant at low [CO_2aq_]. Simulations with the ACTI-CO model of HCO_3_^−^ transport in coccolithophores^14^ were used to evaluate the potential impact of changes in active carbon uptake on *ɛ*_p_ and calculated [CO_2aq_] (Supplementary Methods; Fig. 8). In one set of simulations, we specify a logarithmic dependence of chloroplast HCO_3_^−^ transport/diffusive CO_2_ uptake on [CO_2aq_] as observed in culture experiments^14^,^27^. Alternatively, if enhancement of HCO_3_^−^ transport to the chloroplast is coupled, in part, to reallocation of HCO_3_^−^ from the coccolith vesicle, as inferred from modelling of cultures^14^, our new *ɛ*_coccolith_ and SN coccolith thickness data put additional constraints on the timing of this reallocation. Therefore in a second set of simulations, we specify chloroplast HCO_3_^−^ transport based on HCO_3_^−^ spared from the coccolith vesicle by the reduction in cellular calcite in the last 8 Myr. We then derive the [CO_2aq_] implied by measured *ɛ*_p_ for the specified parameterization of active HCO_3_^−^ uptake to the chloroplast. The results in both cases indicate a greater amplitude of decline in [CO_2aq_] compared with that reconstructed with standard cell size and growth rate considerations only, from around 17 to 6 μM (Fig. 8).

### Calcification in relation to CO_2_ and alkalinity since 1 Myr ago

In the last 1 Myr, climate, the carbon cycle and ocean chemistry underwent significantly higher amplitude variations on glacial–interglacial timescales compared with the preceding 15 Myr. Although our sampling resolution does not capture this higher frequency variability, and does not sample very recent major evolutionary events such as the emergence of *E. huxleyi*, our results nonetheless suggest in the Pleistocene a reversal of the late Miocene–Pliocene trend of more lightly calcified coccoliths and decreasing HCO_3_^−^ allocation to the coccolith vesicle. Within the last 1 Myr, both of these factors rebound to values typical of the late Miocene (Fig. 5). Records based on boron isotopes^36^, alkenone *δ*^13^C (refs 30, 37), and ice cores^38^ suggest *p*CO_2_ values below around 280 p.p.m. over the last 2 Myr, so this increase in degree of calcification contrasts with the generally positive correlation of [CO_2aq_] and degree of calcification observed over the preceding interval.

The change in relationship between degree of coccolith calcification and [CO_2aq_] is even more salient when we examine which samples fall in glacial or interglacial ocean states. Planktic foraminiferal *δ*^18^O values from samples at Indian Ocean Site NGHP-01-01A and the orbital age model for Atlantic ODP Site 925 indicate that our youngest samples at both sites (about 0.27 Myr ago), with high SN coccolith thickness, coincide with glacial periods (Supplementary Fig. 6). The sample at 0.84 Myr ago from Site NGHP-01-01A with high SN coccolith thickness also falls during a glacial period, whereas the sample at 0.95 Myr ago from the Site 925, with lower SN coccolith thickness, falls in an interglacial. These particular sampling points therefore underscore the nature of a change in the relationship between degree of calcification and *p*CO_2_, as the samples with thicker coccoliths in a given size class are from glacial periods that coincide with *p*CO_2_ minima in the last 800 kyr (Supplementary Fig. 6).

In the absence of a coherent relationship with [CO_2aq_], we consider whether a change in ocean alkalinity may have increased cellular HCO_3_^−^ uptake and reduced competition for intracellular HCO_3_^−^, promoting the recovery of degree of coccolith calcification and HCO_3_^−^ allocation to calcification. No proxy record of alkalinity change has yet been produced for this time interval. Multiple lines of evidence based on geochemistry, sedimentology and modelling suggest that the rate of silicate weathering, which adds alkalinity to the ocean, accelerated around 1.5 Myr ago as the North American Precambrian basement shed regolith and experienced more intense subglacial erosion^39^. At the same time the first large amplitude sea level cycles accelerated erosion of shelf sediments^40^. Estimates of alkalinity from carbon system proxies are subject to multiple uncertainties. To explore the magnitude of alkalinity change that might be possible, we compared pH estimates from boron isotopes in planktic foraminifers with estimates of ocean [CO_2aq_] calculated from the cycles of atmospheric *p*CO_2_ recorded in ice cores. While not diagnostic, this analysis suggests the potential for an increase in alkalinity by up to 30% during successive glacials of the last 1.5 Myr (Supplementary Methods; Supplementary Fig. 6). Such an increase would contrast with relatively stable alkalinity inferred for the previous 14 Myr from analysis of the carbonate compensation depth^41^, although such estimates are also subject to multiple uncertainties (Supplementary Fig. 7).

## Discussion

Over the past 14 Myr, selective pressure has acted on the large diversity of different degrees of calcification and morphotypes found in natural coccolithophore populations^15^,^42^,^43^,^44^,^45^,^46^, modifying in a similar way in the tropical Atlantic and Indian Oceans the composition of the population towards better-adapted forms. A similar selective pressure has been suggested for natural populations on seasonal timescales, modulating the relative contribution of different *E. huxleyi* morphotypes with specific degrees of calcification^46^. Long-term mono- and multi-clonal experiments also reveal genotypic selection, as well as beneficial new mutations, as a mechanism for adaptive evolution^11^. Such coccolithophore species or morphotype shifts as a result of ocean changes in the future will arguably have a greater impact on carbon cycle feedbacks than direct physiological responses, highlighting the importance of studying integrated community calcification as well as species- or clone-specific responses^47^,^48^.

The similar long-term decreases in degree of coccolith calcification at our Atlantic and Indian Ocean sites suggest a common selective pressure. This trend in calcification occurs alongside reduced HCO_3_^−^ allocation to the coccolith vesicle. The lack of a coherent relationship between stratification and productivity and SN coccolith thickness at both sites suggest that these factors are not strong candidates to force the common trends in degree of calcification at both sites. In contrast, while the relationship between [CO_2aq_] and cellular calcification has been ambiguous in clonal cell cultures^49^,^50^,^51^, decreasing [CO_2aq_] is one factor shown to reduce HCO_3_^−^ allocation to calcification in modern cells^14^. Changes in [CO_2aq_] are expected to be globally synchronous across the stratified tropical oceans. While the magnitude of [CO_2aq_] decline is sensitive to the inferences about active carbon uptake by algae and detailed steps in [CO_2aq_] cannot be reliably identified given the resolution of our record, a progressive [CO_2aq_] decline since the middle Miocene is evident and correlates with a succession of adaptations in coccolithophore calcification and cell size. A decline in cell size and the first evidence for reduced allocation of HCO_3_^−^ to calcification (decreasing *ɛ*_coccolith_) in the larger coccolithophores occurs several million years before the reduced allocation of HCO_3_^−^ to calcification and reduced cellular calcification in small cells.

The factors driving this differential timing and type of response between the smaller and larger coccolithophores are at this time uncertain but might include lesser plasticity of coccolith thickness in larger genera (*Helicosphaera*, *Coccolithus* and *Calcidiscus*), or a much stronger pressure for HCO_3_^−^ reallocation by larger cells whose diffusive CO_2_ supply was more limited by their low surface area to volume ratio. While decreasing coccolith size has been suggested as one adaptation to decreasing CO_2_ availability^12^,^52^, changes in coccolith size, like changes in degree of calcification or changes in the allocation of available carbon to calcification, appear to be part of an array of possible adaptations that may be used simultaneously or sequentially. These strategies appear to be used to varying degrees in different cell size classes, potentially with different thresholds, as each adaptation may come with its own trade-offs. In addition to varying resource availability documented by geochemical indicators, ‘top down' ecological pressures may contribute to changes in coccolithophore calcification, but unfortunately no proxies are yet available to evaluate their significance in the geological past.

Here, we show a new approach to exploiting independent geochemical and morphological records of coccolithophores to explore the effect of changing cellular HCO_3_^−^ allocation on the magnitude of [CO_2aq_] change inferred from *ɛ*_p_. This approach significantly increases the magnitude of inferred [CO_2aq_] decline over the last 16 Myr, a result that if substantiated by future high-resolution work, would have important implications for our understanding of climate sensitivity.

The coincidence of greatest SN coccolith thickness and inferred degree of cell-calcification with the period of highest [CO_2aq_] in the Miocene is at odds with hypotheses for less-calcified cells under future ocean acidification^15^,^20^,^48^. Similar to future scenarios, proxy records of past pH derived from boron isotopes in planktic foraminifer shells suggest that high Miocene [CO_2aq_] coincided with lower surface ocean pH compared with the Pleistocene (Fig. 7f)^30^,^53^. Although extracellular pH may influence the ease with which protons produced during calcification are exported from coccolithophore cells^54^, and in some culture experiments low pH reduces cellular Particulate Inorganic Carbon (PIC) to Particulate Organic Carbon (POC) ratio and increases the incidence of coccolith malformation (refs 6, 8, 55, 56, 57 but also see refs 7, 58), the limited phenotypic plasticity of short (<20 generations) monoclonal experiments complicates their extrapolation to real-ocean responses.

Consistent with the long-term responses to high [CO_2aq_] and low pH since the Mocene shown here, a recent study in the Bay of Biscay found a dominance of heavily calcified *E. huxleyi* morphotypes during winter when [CO_2aq_] was highest and pH and CaCO_3_ saturation were lowest^46^ (Supplementary Fig. 8). In this study, the low winter calcite saturation state was driven primarily by an increase in dissolved inorganic carbon concentration and, to a minor extent, by reduced temperatures. However, preliminary results for the last 1 Myr suggest that at late Pleistocene [CO_2aq_] levels, increased alkalinity may have favoured a higher degree of cell calcification, consistent with culture experiments at constant [CO_2_], in which increased calcification in *E. huxleyi* accompanied increased alkalinity^6^ (Supplementary Fig. 9). A better understanding of the evolution of ocean alkalinity and [HCO_3_^−^] over the last 1.5 Myr as well as higher-resolution coccolith records may help disentangle the interplay of alkalinity and [CO_2aq_] on coccolithophore calcification. In addition, further studies with natural populations are required to establish whether [CO_2aq_] in the modern ocean is a significant driver of cellular calcification.

The long-term reduction in degree of cell-calcification between 14 and 1 Myr ago could potentially have influenced ocean biogeochemical cycles, if a reduction in coccolith ballast lowered the transfer efficiency of organic carbon to the deep ocean. The reduction in cell calcification we identify is coherent with a recent study documenting a global crash in coccolith CaCO_3_ burial around 4 Myr ago^59^. One consequence of reduced transfer efficiency would be a shallower mean remineralization depth of organic matter. However, *δ*^13^C gradients between surface and thermocline-dwelling foraminifers decrease in par with coccolith thickness from 6 to 4 Myr ago, a change driven primarily by the convergence of thermocline *δ*^13^C values towards surface values (Fig. 6c,d), and suggestive of a deeper depth of organic matter remineralization in the upper water column^60^,^61^. This trend suggests that the effect of global cooling, which acts to slow remineralization rates, overrode any effect of reduced ballasting and led to a deepening of mean remineralization depth during the late Miocene^60^. If future global warming likewise leads to a shoaling of mean remineralization depth, it may act to counteract any shift towards enhanced ballasting by more heavily calcified coccolithophore cells.

In summary, our observations suggest that on long timescales, increased [CO_2aq_] and increased alkalinity may contribute selective pressures favouring thicker coccoliths of a given size and a higher degree of cell-calcification. As projected changes in surface ocean chemistry simulate increased [CO_2aq_] but diminished alkalinity, prediction of the sign of calcification will rely on better defining the thresholds of response to each parameter. In addition, it remains to be determined whether coccolithophore responses to rapid ocean chemistry changes in the future will be analogous to the geological-timescale adaptation studied here. The plasticity of coccolith thickness and potential selective pressures in the genetically diverse modern ocean thus warrant further investigation.

## Methods

### Cellular calcification and thickness in culture

Eight clonal strains of *E. huxleyi* and *Gephyrocapsa* (Supplementary Table 1) were maintained as dilute batch cultures in natural seawater from the Cantabrian Sea (Northern Spain). Prior to experiments, seawater was sterile filtered at 0.2 μm, heated to 80 °C for 3 h, cooled overnight in a sterile hood, and pH was adjusted to 8.3 by addition of NaOH. Media was enriched with major nutrients (P, N), trace metals and vitamins according to the K/2 recipe^62^ modified by eliminating the Tris buffer and silicate. Media was then sterile filtered at 0.2 μm just prior to inoculation. Experiments were carried out under a light:dark cycle of 16:8 h at a constant temperature of 16 °C under saturated light growth conditions (80–150 μmol m^−2^ s^−1^ photon flux). A homogeneous distribution of cells was maintained by placing the cultures on a roller system providing gentle rotation during growth. Through serial dilution, cells were maintained in dilute cultures for 8–12 generations before sampling, to establish stable nutrient and carbon chemistry in the media. Each strain was grown in duplicate, and in some cases triplicate, culture bottles. One strain was cultured in two separate experiments and each experiment is reported separately because the two experiments had opposite trends of drift in pH. On collection, media pH and total alkalinity were measured with a Crison GLP-21pH metre calibrated with National Bureau of Standards (NBS) buffers and quadruplicate potentiometric titration of filtered, poisoned media samples on a Crison TitroMatic 1S, respectively^63^,^64^. Average media alkalinity was 2,572 μmol kg^−1^ (±115, 1 s.d.), pH 8.22 (±0.07, 1 s.d.) with drift in pH during experiments of <0.11 pH units. Cell density was maintained at biomass averaging 1.65 μg C ml^−1^ and in all cases <2.5 μg C ml^−1^. Cell counts were determined at harvest with a Fuchs–Rosenthal haemocytometer.

For determination of cellular carbon quota, cells were harvested on pre-combusted GF/F or QFF filters. Following acidification to remove calcite, they were analysed for carbon content by flash combustion EA (Euro Vector EA-1108) at 1,020 °C coupled with a Nu Instruments mass-spectrometer (Nu-Horizon). For determination of cellular calcite, cells were harvested on polycarbonate filters. Filters were acidified in 2% HNO_3_ and Ca concentration was measured in the resultant solutions by ICP-AES (Thermo ICAP DUO 6300).

Cell size (radius) and cell surface area (SA) were derived from measurements of cellular carbon quota using the regression of Popp *et al.*^65^, which is similar to those derived from other studies^66^,^67^. We use PIC/SA as an optimal way to represent calcification across a range of cell sizes, because it is unaffected by the size scaling of surface area/volume (SA/V). The PIC/POC ratio is often used to describe the degree of calcification per cell, but scales with SA/V, which is dependent on cell size. In published culture studies, at the onset of the photoperiod and just after division, when cell size is smallest and SA/V is highest, the PIC/POC ratio is 30–36% higher whereas PIC normalized to cell surface area is only 10–12% higher^68^. For a given strain of coccolithophore, this inverse correlation between cell size and PIC/POC ratio is widespread in published culture studies (Supplementary Fig. 10) because of the large plasticity of cell size in response to changes in light regime, carbonate system parameters, and temperature. In our culture samples, because of the limited range in cell size, and larger range in coccolith morphology across different strains, both PIC/SA and PIC/POC yield similar results (Supplementary Fig. 1, Supplementary Table 2).

For determination of coccolith mass and thickness, cells were collected on polycarbonate filters. Coccoliths were extracted from the filters by addition of 0.5 ml ethanol with gentle ultrasonication, and evaporation of the ethanol suspension on a glass microscope slide. This ensured that coccoliths were in a single plane of focus for polarized microscopy. A total of 25–55 random fields of view (FOV) were imaged from microscope slides using a Nikon DS-Fi1 8-bit colour digital camera, Nis-Elements software and a Nikon Eclipse LV100 POL polarized light microscope equipped with a × 100 H/N2 objective set-up with circular polarization at Salamanca University (resultant area of one pixel=0.035 μm^2^). For full details of the circular polarization microscope set-up applied in this study, see ref. 69. The measurement of coccolith thickness from birefringence relies on the systematic relationship between the thickness of a calcite particle and the interference colour that it displays under polarized light^69^,^70^,^71^. In the thickness range 0–1.55 μm, calcite particles show first-order polarization colours ranging from black to white. Calcite particles thicker than 1.55 μm display first-order polarization colours ranging from yellow through to pink up to a thickness of 4.5 μm, beyond which second-order colours are observed^71^. For culture samples, we use a calibration based on a calcite wedge of known thickness to convert grey level to thickness. Images were processed with C-Calcita^69^. All coccoliths were analysed with a minimum of 100 per sample, and coccolith length, width and area were measured.

### Sites and age models

**Figure 1:**
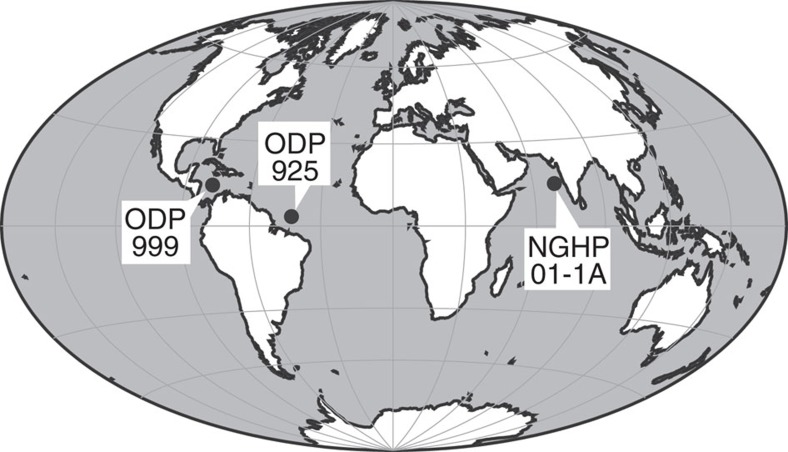
Map showing site locations.

**Figure 2:**
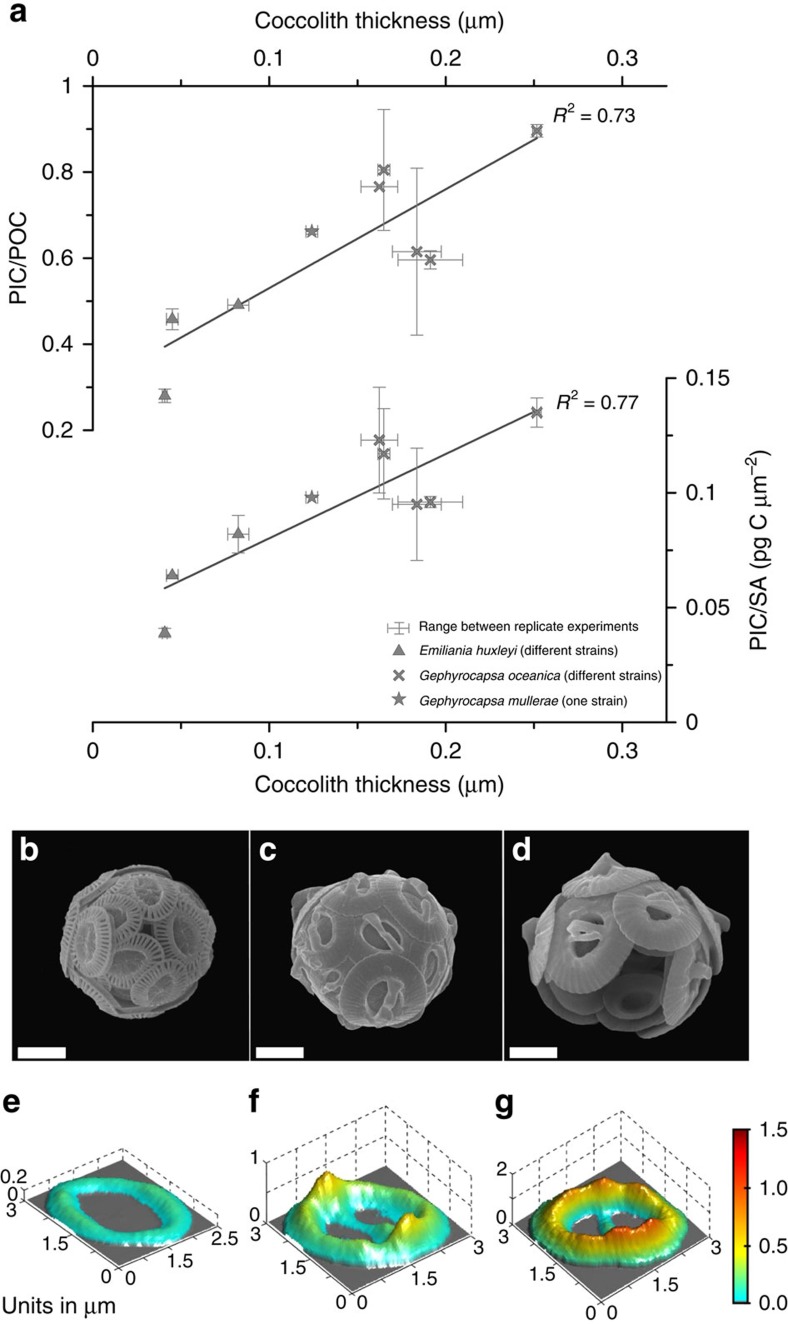
Noëlaerhabdaceae coccolith morphology in culture. (**a**) Relationship between coccolith thickness and cellular PIC/POC (particulate inorganic carbon/particulate organic carbon) and cellular PIC/cell SA (surface area) for modern strains of *Emiliania huxleyi* and *Gephyrocapsa* grown in laboratory culture (Supplementary Table 1). Symbols are averages for each experiment and lines show the range of values between replicate culture bottles for each experiment. Scanning electron microscope images of coccospheres from the strains with lowest (RCC 1257, **b**), intermediate (RCC 3370, **c**) and highest (RCC 1292, **d**) coccolith thickness. Scale bar, 2 μm (in all images). (**e**–**g)** Three-dimensional representations of coccolith thickness for the same strains as coccospheres. The vertical scale shows cumulative thickness from zero at the base; therefore in the central area of *Gephyrocapsa* coccoliths, the bridge (central area bar) is displaced towards the base of the plane of illustration.

Fifteen samples spanning the last 14 Myr ago were selected from two low-latitude sites to investigate the evolution of coccolith calcification: ODP Site 925 in the western tropical Atlantic Ocean (4°12.248′ N, 43°29.349′ W, water depth 2041, m) and Site NGHP-01-01A in the eastern Arabian Sea (Kerala–Konkan Basin, 15°18.366′ N, 070° 54.192′ E, water depth 2663, m; Fig. 1). Late Neogene sedimentary sections at both sites are primarily calcareous ooze, and sites were selected on the basis of their good coccolith preservation. The age model for Site NGHP-01-01A is based on calcareous nannofossil biostratigraphy^72^, and ODP Site 925 ages are based on astronomical calibration of shipboard physical property data (ref. 73; S.J. Crowhurst and H. Pälike, personal communication, 2013).

### Coccolith mass and size measurements in sediments

**Figure 3:**
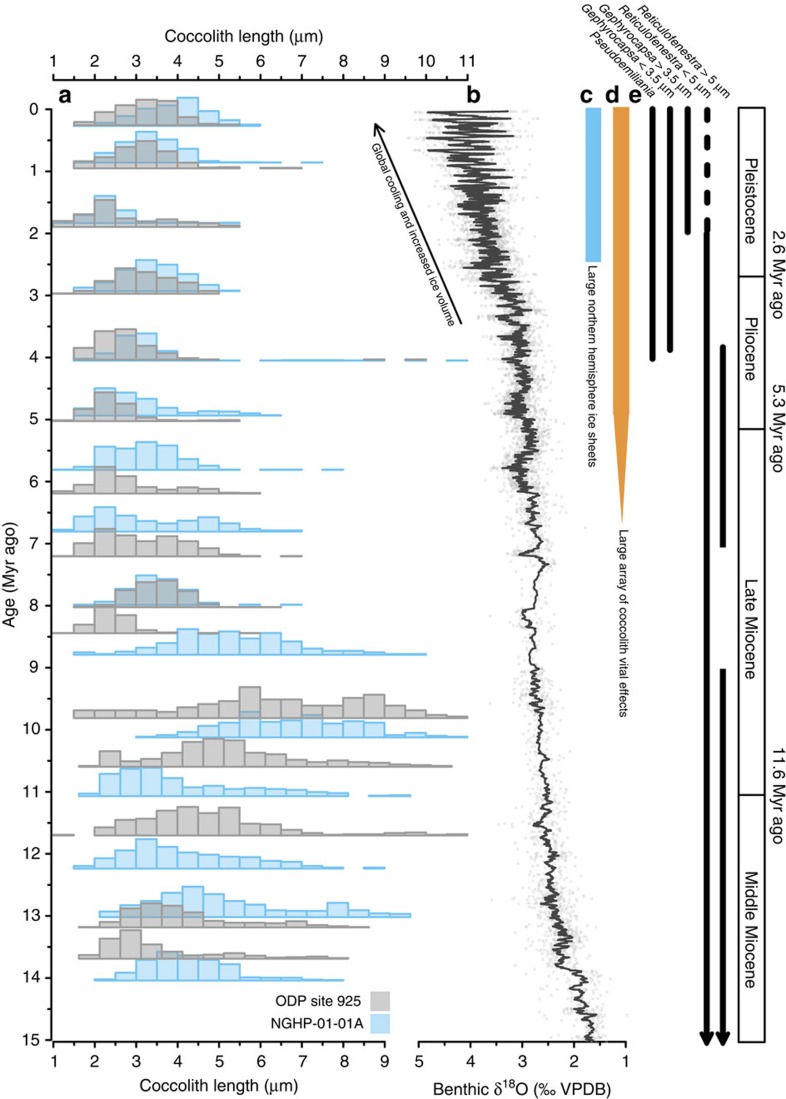
Long-term evolution of Noëlaerhabdaceae coccolith size and stable isotope vital effects with climate over the last 14 Myr. (**a**) Noëlaerhabdaceae coccolith size distributions over time at Sites ODP 925 (grey) and NGHP-01-01A (blue). (**b**) Climate evolution over the Neogene represented by a benthic foraminiferal *δ*^18^O stack (data compiled by ref. 86). (**c**) Onset of major northern hemisphere glaciation at ∼2.6 Myr ago. (**d**) The emergence of large scale vital effects in the *δ*^18^O and *δ*^13^C of coccolith calcite around 7–5 Myr ago (ref. 14; this study). (**e**) Approximate age ranges of Neogene genera belonging to the Noëlaerhabdaceae family^76^.

**Figure 4:**
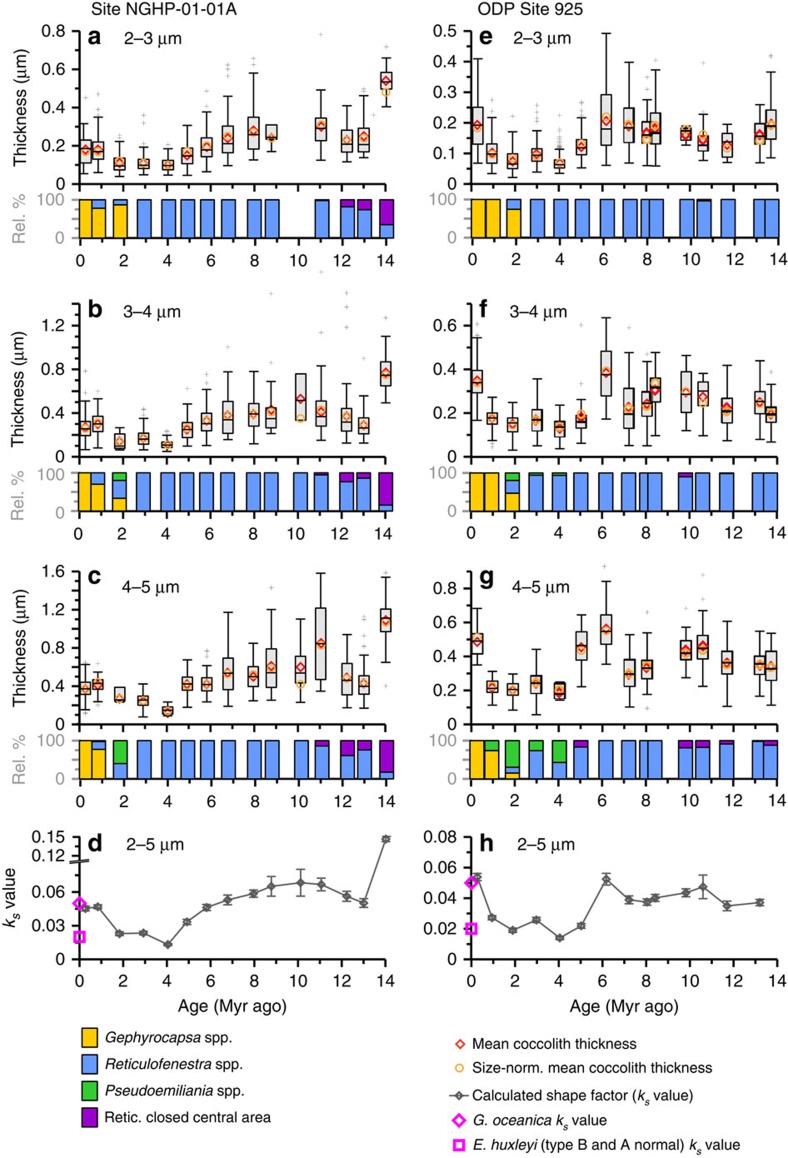
Changes in Noëlaerhabdaceae coccolith thickness and *k*_s_ value at two tropical sites since 14 Myr ago. (**a**–**d**) Site NGHP-01-01A, and (**e**–**h**) ODP Site 925. (**a**–**c**,**e**–**g**) Thickness data for coccoliths of 2–3, 3–4 and 4–5 μm length. Box–Whisker plots illustrate coccolith thickness data for each sample and size class (box shows median value and upper/lower quartiles, whiskers show maximum and minimum values, outliers >1.5 × the interquartile range are shown as crosses). Also shown are mean values of raw (circles) and SN (diamonds) thickness (Supplementary Data 1). Bar graphs show the relative contribution of different genera to the Noëlaerhabdaceae population in each size class and sample. (**d**,**h**) *k*_s_ values (error bars are ±2 s.e.m.). The shape factor *k*_s_, which expresses the fraction of the volume of a cube defined by the length of a coccolith that is composed of biomineral^77^, was originally proposed to estimate coccolith mass from coccolith length and is similar to coccolith thickness. However, unlike thickness, *k*_s_ does not account for variations in coccolith circularity. Pink symbols are *k*_s_ for extant Noëlaerhabdaceae species^77^.

**Figure 5:**
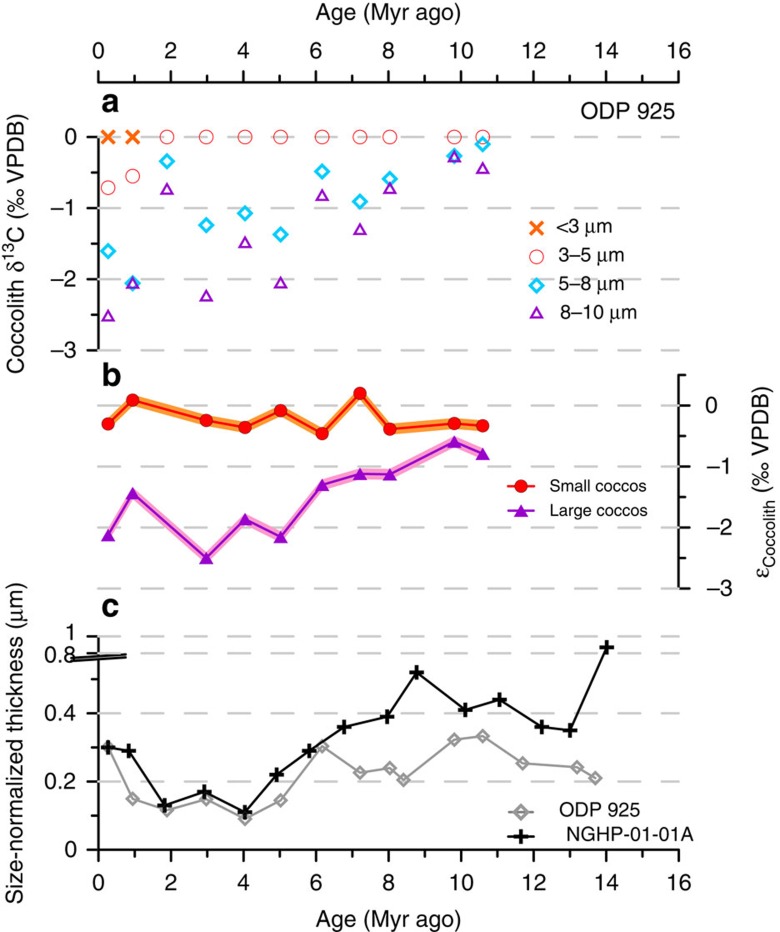
Coccolith geochemistry and SN thickness trends since the Miocene. (**a**) *δ*^13^C values for size-separated coccoliths from ODP Site 925, normalized to the smallest coccolith size fraction. (**b**) *ɛ*_coccolith_ for small (3–5 μm) and large (8–10 μm) coccoliths from Site 925. Shading indicates propagated analytical uncertainty on *δ*^13^C measurements. (**c**) mean SN coccolith thickness for Noëlaerhabdaceae coccoliths of 2–5 μm lengths at ODP Site 925 and Site NGHP-01-01A (coccolith thicknesses are normalized to mean coccolith length within the 2–5-μm size fraction over the whole time series at each site, that is, 3.52 and 3.93 μm, respectively).

**Figure 6:**
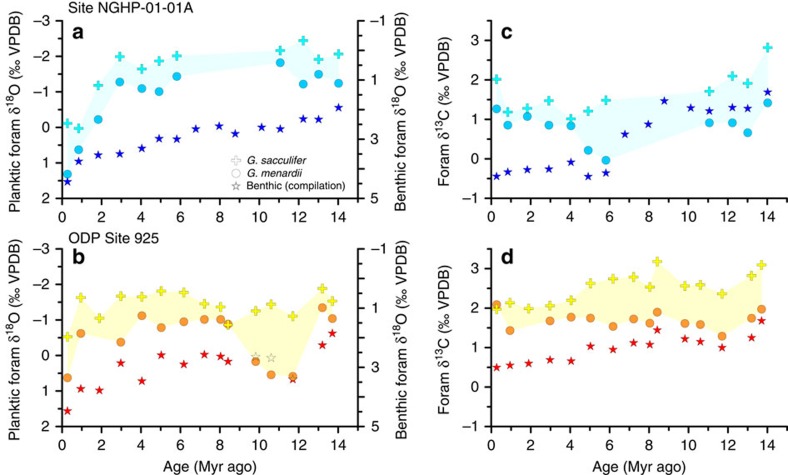
Foraminiferal stable isotope records as indicators of water column structure. *δ*^18^O (**a**,**b**) and *δ*^13^C (**c**,**d**) records for surface (*G. sacculifer*), thermocline (*G. menardii*) and benthic species at Sites ODP 925 and NGHP-01-01A. Benthic isotope data for our sites are not available, therefore values were interpolated using a global compilation^86^ separated into ocean basins. Because Neogene Indian Ocean data are sparse and trends are very similar to those in the Pacific, a compilation of Indian and Pacific Ocean data were used to interpolate the benthic values in **a** and **c**. For **b** and **d**, a compilation of all Atlantic data was used. Note: benthic *δ*^18^O is plotted on a different *y* axis. Shading indicates the gradient between surface and thermocline-dwelling planktic foraminiferal species.

**Figure 7:**
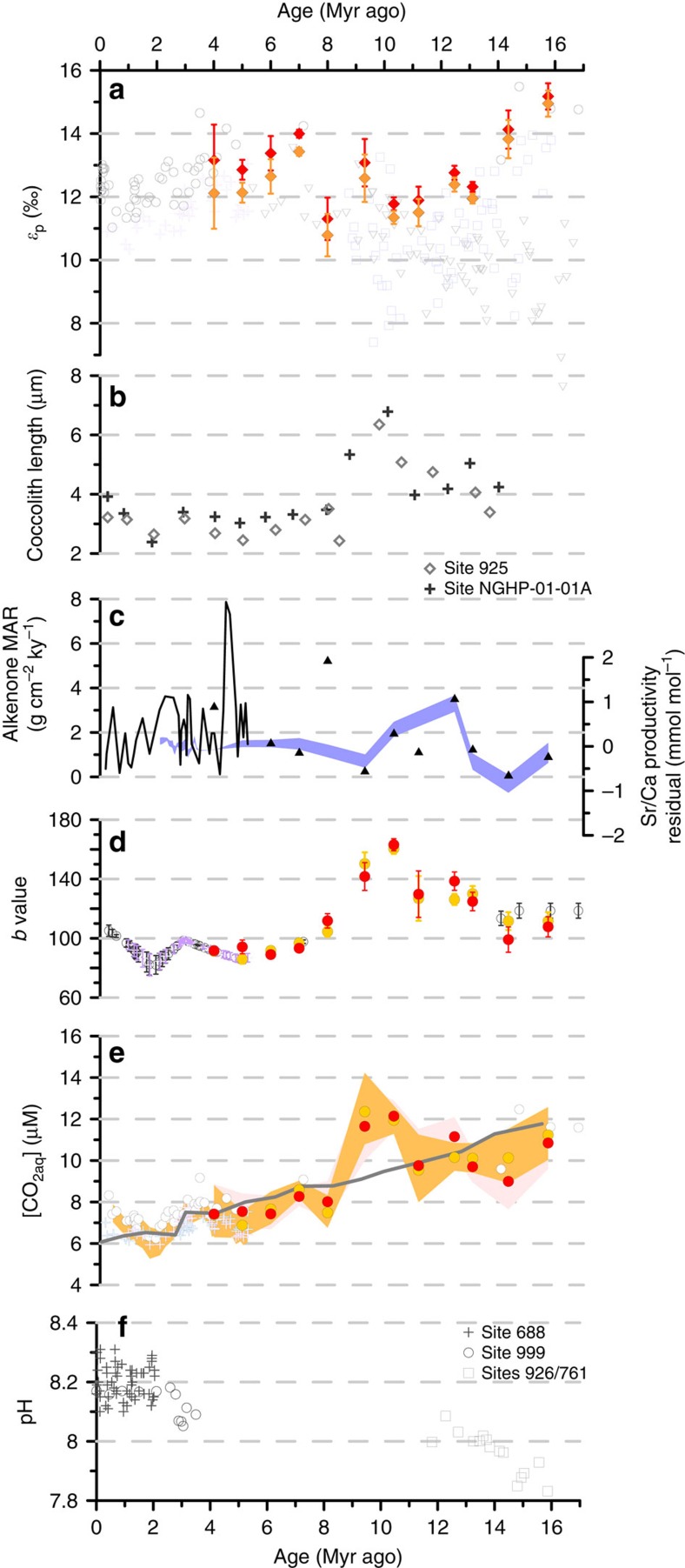
*ɛ*_p_ values and estimates of *b* and [CO_2aq_] at Caribbean ODP Site 999 and other sites for the last 17 Myr. (**a**) New *ɛ*_p_ data (Site 999, red diamonds: SST max, orange diamonds: SST min; error bars show propagated analytical uncertainty on *δ*^13^C measurements). Published *ɛ*_p_ records: Site 999 (ref. 30) (purple crosses), ODP Site 925 (ref. 37) (grey circles), DSDP Sites 588 (ref. 84) (grey triangles, maximum *ɛ*_p_) and 608 (ref. 84) (blue squares). (**d**) Variations in *b* (Site 999) inferred to arise from changing cell size (**b**) and growth rate (**c**) (see Methods). In **c**, triangles (this study) and line^30^ show alkenone MARs and blue shading shows Sr/Ca productivity estimates for small coccolithophores^14^ (all Site 999). In **d**, purple crosses (Site 999 (ref. 30)), grey circles (Site 925 (ref. 37)), and orange circles (Site 999, this study) show *b* values recalculated using our new cell size correction. Red circles (Site 999, this study) show *b* values calculated with cell size and growth rate corrections. For error calculations, see Methods. (**e**) [CO_2aq_] calculated using cell size (orange circles), or cell size plus growth rate (red circles), correction and *ɛ*_p_ values (Site 999, this study). [CO_2aq_] was also recalculated using our cell size correction for the Plio-Pleistocene at Site 999 (ref. 30) (purple crosses) and Site 925 (ref. 37) (grey circles). For all sites, reference *b*=150. [CO_2aq_] assuming constant *b* for Site 999 (ref. 30) is also shown (blue crosses). Shading indicates maximum and minimum [CO_2aq_] estimates for all data from Site 999 (see Supplementary Methods). We do not apply our size correction to DSDP Sites 608 and 588 *ɛ*_p_ data because these sites are at significantly higher latitudes; therefore cell size history may be different compared with the tropical sites studied here. Also shown in **e** is the [CO_2aq_] expected for the Caribbean site if it were in equilibrium with the atmospheric *p*CO_2_ modelled by ref. 33 (grey line). (**f**) pH derived from *δ*^11^B of planktic foraminifers, for the Plio-Pleistocene^30^,^36^ and Miocene^53^. During the Miocene, ODP Site 999 *ɛ*_p_ values are similar to values at ODP Site 925 (ref. 37) and higher than values from DSDP Sites 588 and 608 (ref. 85). From 16 to 9 Myr ago, the maximum *ɛ*_p_ at DSDP Site 608 shows a similar trend to *ɛ*_p_ at ODP Site 999, albeit with slightly lower absolute values, suggesting that either both sites experienced similar changes in growth rates, or that a global CO_2_ component exerted a dominant forcing on both *ɛ*_p_ records. The temporally variable scatter to low *ɛ*_p_ values seen in the Site 608 record may result from higher frequency oscillations in growth rates at this site^83^. The much lower average *ɛ*_p_ at Site 588 suggest that this site experienced on average higher phytoplankton growth rates and productivity compared to Sites 925, 999 and 608.

**Figure 8:**
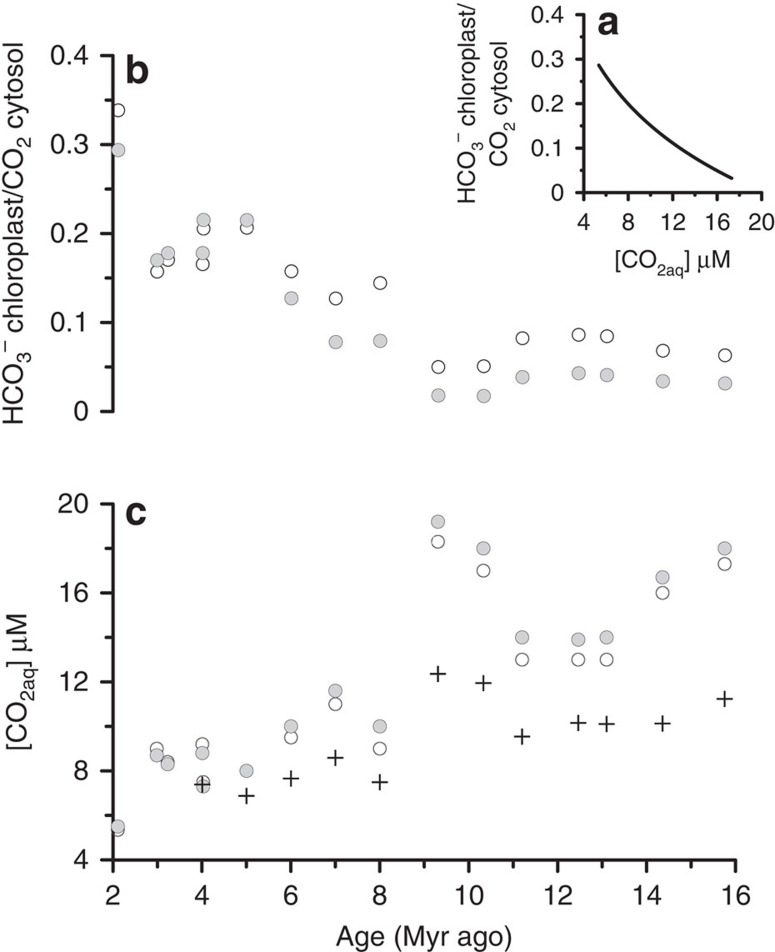
Simulations of the effect of active HCO_3_^−^ uptake on reconstructed [CO_2aq_]. ACTI-CO model simulation for two potential scenarios of active HCO_3_^−^ uptake to the chloroplast (**b**), and consequences for [CO_2aq_] implied by alkenone *ɛ*_p_ measurements (**c**). A first simulation (unfilled circles) employs a logarithmic dependence of HCO_3_^−^ transport to the chloroplast on [CO_2aq_], similar to that observed in cultures (**a**). A second simulation (filled circles) supplements HCO_3_^−^ supply to the chloroplast as a function of HCO_3_^−^ spared from the coccolith vesicle by the production of thinner coccoliths (that is, a reduced PIC per cell surface area). Crosses in **c** show [CO_2aq_] estimated from standard regressions between *ɛ*_p_ and [CO_2aq_] as shown in Fig. 7e (orange circles).

Microscope slides for image analysis were prepared using a quantitative decantation method producing a homogenous distribution of coccoliths^74^. A total of 20–50 random FOV were imaged as described above for cultures. Microscope light settings and camera parameters were kept constant during each imaging session, and calibration images of the same set of calcite particles (the same *Rhabdosphaera* coccoliths in our youngest ODP Site 925 Pleistocene sample) were taken at the start of each session to account for bulb ageing and the different light requirements for different sample groups. Because the method applied here uses grey scale images to estimate coccolith thickness, it is only applicable to coccoliths thinner than 1.55 μm. In the late Miocene, some large Noëlaerhabdaceae coccoliths display yellow or orange first-order interference colours. For this reason, although size data are presented for all Noëlaerhabdaceae coccoliths present in our samples (Fig. 3), we present only mass and thickness measurements for coccoliths in the length range 2–5 μm, which exhibit only grey scale colours throughout the time series (Fig. 4). All whole Noëlaerhabdaceae coccoliths were analysed with a minimum of 300 per sample. Coccolith length, width and area were also measured. Coccolith mass values in this study are comparable to published data using similar birefringence-based methods (Supplementary Fig. 11). To isolate the component of variation in coccolith thickness that represents a change in calcification and does not occur as a direct result of changes in coccolith size, we (1) examine changes in thickness within narrow size classes (2–3, 3–4 and 4–5 μm), and (2) calculate SN thickness of coccoliths within each size classes to further verify that changes in thickness are not a direct result of size changes (that is, the expectation that a larger coccolith is thicker as a result of isometric scaling). Following ref. 75, we use the equation:


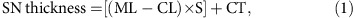


where ML is the mean coccolith length within the size fraction in question over the whole time series at a given site (see Supplementary Data 1 for values used), CL is the length of coccolith X in Sample A, *S* is the slope of the regression between coccolith length and coccolith thickness for all coccoliths in Sample A and CT is the original thickness of coccolith X in Sample A.

### Coccolith taxonomy and preservation

We identified coccoliths to genus level only because species-level classification of the smallest Noëlaerhabdaceae can be difficult under the light microscope, and because commonly used *Reticulofenestra* species assignations are primarily based on size^76^. Although most coccoliths were complete, some were found to be missing a piece of the outer rim cycle of one or both shields when observed under the SEM. To verify that such fragmentation did not result in underestimation of coccolith mass per unit area, we quantify this potential effect from SEM images of the 2–5 μm fraction of fossil samples and estimated the percentage of mass loss for individual Noëlaerhabdaceae coccoliths due to fragmentation (minimum 50 coccoliths per sample) using C-Calcita. In these calculations, we assumed that 50% of the total mass of each coccolith comes from the inner rim cycle and central area structure (bridge and/or grill), and that the outer rim cycle of each shield contributed 25% of the total mass. These assumptions were based on mass analyses of very well-preserved modern water column samples containing a mixture of *Gephyrocapsa* and *E. huxleyi* coccoliths. In these samples, mean contribution of inner rim plus central area to total mass was 63±10% (1 s.d.), therefore our choice of 50% for fossil Noëlaerhabdaceae calculations is conservative.

Coccolith dimensions are used to infer cell size, based on relationships between coccolith length and cell size for Noelaerhabdaceae^13^,^52^. On this basis, we attribute geochemical results from large coccoliths (8–10 μm) as those characterizing larger cells, and those of smaller coccoliths (3–5 μm) as those characterizing small cells. *k*_s_ values (originally devised to estimate mass from coccolith shape), were calculated from fossil coccolith mass and length data using the equation of ref. 77:


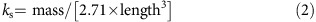


We verify that temporal patterns in Noëlaerhabdaceae coccolith mass and thickness result only from primary biomineralization and not from abiogenic post-depositional overgrowth using qualitative preservation indices and SEM images (see Results, Supplementary Data 1; Supplementary Figs 4 and 5). Noëlaerhabdaceae with a closed central area occur in some samples older than 10 Myr ago at both sites, and because of the presence of some overgrowth near the central area in some specimens, we remain cautious in always identifying this as a primary morphological feature. Almost all *Reticulofenestra* in the 2–5 μm fraction of our 14 Myr ago sample at Site NGHP-01-01A have closed central areas and this appears to be a primary feature (Supplementary Fig. 5s,t), resulting in very high mean SN thickness and *k*_s_ values in this sample (Fig. 4). *Cyclicargolithus* coccoliths were seen sporadically in our oldest samples, although these were all >5 μm.

### Assumptions related to coccoliths per cell

Estimation of changing cellular calcite/SA from coccolith thickness requires that cellular calcite/SA be regulated more strongly by coccolith thickness than by the number of coccoliths per cell. Modern Noëlaerhabdaceae coccolithophore cells are typically covered with a monolayer comprised of interlocking coccoliths, with the exception of *E. huxleyi* that has a higher tendency to produce multi-layered coccospheres^78^. The number of coccoliths per cell has been shown to vary with the cell division cycle of a coccolithophore, with the accumulation of extra coccoliths immediately before cell division to ensure adequate coverage of the two daughter cells^68^. Recent work in culture^79^ has confirmed that stationary phase cells, which are essentially paused in the stage just prior to cell division due to lack of nutrients, are more often covered with a higher number of coccoliths compared with exponentially growing cells. While natural populations in the ocean contain cells at a variety of life stages, that is, not synchronized in the same way as a culture population, populations of *Coccolithus pelagicus* recovered from the surface ocean during the North Atlantic spring bloom still have on average around 20–25% fewer coccolith per cell than populations growing in non-bloom conditions, analogous to in culture^79^. These differences may be useful in characterizing major swings in productivity in fossil populations, in the rare cases where exceptional preservation conserves a large number of intact coccospheres. Quantification of changes in mean number of coccoliths per cell in fossil populations is not possible in this study because it requires significant numbers of whole coccospheres to be preserved, a phenomenon that is rare in deep, open-ocean sites such as ours (selected to be representative of global change) and on the long timescales at which we are working at a single site. However, in sediment populations such as those in our study, which integrate a time window of a few hundred to a thousand years (taking into account sedimentation rates and bioturbation), seasonal and inter-annual productivity changes that might cause higher representation of one growth phase versus another and concomitant changes in coccoliths per cell are likely averaged out. In addition, export of phytoplankton to the sediments is typically biased towards high production periods, so the significance of true stationary phase growth to export and the sediment population is likely diminished.

### Stable isotopes in foraminifers

Isotope data for two planktic foraminiferal species at Sites NGHP-01-01A and ODP Site 925 were generated. Approximately 25 individuals of *G. sacculifer* (without sac-like final chamber) and *G. menardii* were picked from the 250–350-μm and the 300–400-μm size fractions, respectively. Foraminifers were broken open and ultrasonicated in methanol to remove fine fraction contamination, rinsed with MilliQ water, dried at 55 °C, and analysed on a Nu Instruments Perspective DI-IRMS connected to an automated carbonate preparation system (Nu Carb), with an analytical precision of 0.06‰ for *δ*^18^O and 0.05‰ for *δ*^13^C (1σ), at Oviedo University.

The variable depth habitats of planktic foraminifers allow us to reconstruct changes in upper water column properties via oxygen and carbon isotopic gradients. In tropical open-ocean settings such as those overlying our two sites, *G. sacculifer* is thought to live and calcify in the upper mixed layer of the ocean, whereas *G. menardii* favours the upper-middle thermocline^24^,^25^,^80^,^81^. To a first order, foraminiferal *δ*^18^O gradients between the upper mixed layer and thermocline in well-stratified regions of the tropical ocean reflect the upper photic zone temperature and salinity gradients^24^, although some additional physiological (vital effects) and environmental (for example, carbonate ion concentration) factors also affect isotopic fractionation^81^. Foraminiferal *δ*^13^C gradients between the upper mixed layer, the thermocline and the deep ocean at both sites were used to evaluate the depth of organic matter remineralisation, following refs 60, 61. Planktic foraminiferal *δ*^13^C values were corrected to dissolved inorganic carbon (DIC) following ref. 81.

### Stable isotopes in coccoliths

Samples were disaggregated and micro-filtered in 2% ammonia to separate coccolith size fractions (<3, 3–5, 5–8 and 8–10 μm), which were rinsed three times with MilliQ water and dried at 55 °C. All coccolith fractions were examined under the microscope, and fractions from Site NGHP-01-01A were found to be heavily contaminated with fragments of non-coccolith carbonate. For this reason, isotope data for these samples are not presented or considered further. At ODP Site 925, coccolith size fractions contain solely coccolith carbonate. In samples older than 3 Myr ago, coccolith fragments contribute significantly to the <3 μm fraction so data points were excluded. Coccolith samples were analysed as described above for foraminifers. Mean reproducibility, based on duplicate analyses of splits of 22 random coccolith samples from Site 925, is 0.08‰ for *δ*^18^O and 0.07‰ for *δ*^13^C (1σ). *ɛ*_coccolith_ of small and large coccoliths was calculated from coccolith *δ*^13^C relative to *G. menardii δ*^13^C because this foraminiferal species calcifies in equilibrium with *δ*^13^C DIC^81^, and also has a similar depth habitat to the coccolithophores with maximum abundances in the chlorophyll maximum near the thermocline^82^; such that


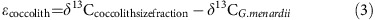



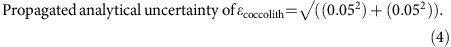


For one sample around 2 Myr ago, there was an insufficient number of *G. menardii* individuals for analysis, therefore we were unable to calculate *ɛ*_coccolith_.

See Supplementary Methods for details of carbon isotope determinations in alkenones, *ɛ*_p_ and [CO_2aq_] calculations, and details on ACTI-CO simulations to quantify the effect of changing active uptake on [CO_2aq_] estimates.

## Additional information

**How to cite this article**: Bolton, C. T. *et al.* Decrease in coccolithophore calcification and CO_2_ since the middle Miocene. *Nat. Commun.* 7:10284 doi: 10.1038/ncomms10284 (2016).

## Supplementary Material

Supplementary Figures and Supplementary Tables Supplementary Figures 1-13 and Supplementary Tables 1-3, Supplementary Methods, Supplementary References

Supplementary Data 1 Coccolith thickness, isotope, size, and preservation data

Supplementary Data 2 Alkenone, ep, and Sr/Ca productivity data at ODP Site 999

## References

[b1] Klaas C. & Archer D. E. Association of sinking organic matter with various types of mineral ballast in the deep sea: implications for the rain ratio. Global Biogeochem. Cycles 16, 1–14 (2002).

[b2] Hain M., Sigman D. & Haug G. in Treatise on Geochemistry 2nd edn Vol. 8(18), eds Mottl M. J., Elderfield Henry 485–517 (2013).

[b3] Feely R. A. *et al.* Impact of anthropogenic CO_2_ on the CaCO_3_ system in the oceans. Science 305, 362–366 (2004). 15256664 10.1126/science.1097329

[b4] Rost B., Zondervan I. & Wolf-Gladrow D. Sensitivity of phytoplankton to future changes in ocean carbonate chemistry: current knowledge, contradictions and research directions. Mar. Ecol. Prog. Ser. 373, 227–237 (2008).

[b5] Bach L. T., Bauke C., Meier K. J. S., Riebesell U. & Schulz K. G. Influence of changing carbonate chemistry on morphology and weight of coccoliths formed by *Emiliania huxleyi*. Biogeosciences 9, 3449–3463 (2012).

[b6] Bach L. T. *et al.* Dissecting the impact of CO_2_ and pH on the mechanisms of photosynthesis and calcification in the coccolithophore *Emiliania huxleyi*. New Phytol. 199, 121–134 (2013). 23496417 10.1111/nph.12225

[b7] Jones B. M. *et al.* Responses of the *Emiliania huxleyi* proteome to ocean acidification. PloS One 8, e61868 (2013). 23593500 10.1371/journal.pone.0061868 PMC3625171

[b8] Langer G. *et al.* Species-specific responses of calcifying algae to changing seawater carbonate chemistry. Geochem. Geophys. Geosyst. 7, Q09006 (2006).

[b9] Langer G., Nehrke G., Probert I., Ly J. & Ziveri P. Strain-specific responses of *Emiliania huxleyi* to changing seawater carbonate chemistry. Biogeosciences 6, 2637–2646 (2009).

[b10] Jin P., Gao K. & Beardall J. Evolutionary responses of a coccolithophorid *Gephyrocapsa oceanica* to ocean acidification. Evolution 67, 1869–1878 (2013). 23815645 10.1111/evo.12112

[b11] Lohbeck K. T., Riebesell U. & Reusch T. B. Adaptive evolution of a key phytoplankton species to ocean acidification. Nat. Geosci. 5, 346–351 (2012).

[b12] Hannisdal B., Henderiks J. & Liow L. H. Long-term evolutionary and ecological responses of calcifying phytoplankton to changes in atmospheric CO_2_. Global Change Biol. 18, 3504–3516 (2012).

[b13] Henderiks J. Coccolithophore size rules - reconstructing ancient cell geometry and cellular calcite quota from fossil coccoliths. Mar. Micropaleontol. 67, 143–154 (2008).

[b14] Bolton C. T. & Stoll H. M. Late Miocene threshold response of marine algae to carbon dioxide limitation. Nature 500, 558–562 (2013). 23985873 10.1038/nature12448

[b15] Beaufort L. *et al.* Sensitivity of coccolithophores to carbonate chemistry and ocean acidification. Nature 476, 80–83 (2011). 21814280 10.1038/nature10295

[b16] Meier K. J. S., Beaufort L., Heussner S. & Ziveri P. The role of ocean acidification in *Emiliania huxleyi* coccolith thinning in the Mediterranean Sea. Biogeosciences 11, 2857–2869 (2014).

[b17] Horigome M. T. *et al.* Environmental controls on the *Emiliania huxleyi* calcite mass. Biogeosciences 11, 2295–2308 (2014).

[b18] Meier K. J. S., Berger C. & Kinkel H. Increasing coccolith calcification during CO_2_ rise of the penultimate deglaciation (Termination II). Mar. Micropaleontol. 112, 1–12 (2014).

[b19] Gibbs S. J., Robinson S. J., Bown P. R., Dunkley Jones T. & Henderiks J. Comment on "Calcareous nannoplankton response to surface-water acidification around Oceanic Anoxic Event 1a". Science 332, 175 (2011). 21474738 10.1126/science.1199459

[b20] Riebesell U. *et al.* Reduced calcification of marine plankton in response to increased atmospheric CO_2_. Nature 407, 364–367 (2000). 11014189 10.1038/35030078

[b21] Read B. A. *et al.* Pan genome of the phytoplankton *Emiliania* underpins its global distribution. Nature 499, 209–213 (2013). 23760476 10.1038/nature12221

[b22] Kameo K. & Bralower T. J. in Proceedings of the Ocean Drilling Program. Scientific Results 165, eds Leckie R. M., Sigurdsson H., Acton G. D., Draper G. 3–17Ocean Drilling Program (2000).

[b23] Young J. R. Size variation of Neogene *Reticulofenestra* coccoliths from Indian Ocean DSDP cores. J. Micropalaeontol. 9, 71–85 (1990).

[b24] Steph S., Regenberg M., Tiedemann R., Mulitza S. & Nürnberg D. Stable isotopes of planktonic foraminifera from tropical Atlantic/Caribbean core-tops: Implications for reconstructing upper ocean stratification. Mar. Micropaleontol. 71, 1–19 (2009).

[b25] Chaisson W. & Ravelo A. In Proceedings of the Ocean Drilling Program. Scientific Results 255–268Ocean Drilling Program (1997).

[b26] Rost B., Zondervan I. & Riebesell U. Light-dependent carbon isotope fractionation in the coccolithophorid *Emiliania huxleyi*. Limnol. Oceanogr. 47, 120–128 (2002).

[b27] Cassar N., Laws E. A. & Popp B. N. Carbon isotopic fractionation by the marine diatom *Phaeodactylum tricornutum* under nutrient- and light-limited growth conditions. Geochim. Cosmochim. Acta 70, 5323–5335 (2006).

[b28] Gupta A. K., Singh R. K., Joseph S. & Thomas E. Indian Ocean high-productivity event (10–8 Ma): linked to global cooling or to the initiation of the Indian monsoons? Geology 32, 753–756 (2004).

[b29] Diester-Haass L., Billups K. & Emeis K. C. In search of the late Miocene-early Pliocene “biogenic bloom” in the Atlantic Ocean (Ocean Drilling Program Sites 982, 925, and 1088). Paleoceanography 20, (2005).

[b30] Seki O. *et al.* Alkenone and boron-based Pliocene *p*CO_2_ records. Earth. Planet. Sci. Lett. 292, 201–211 (2010).

[b31] Pagani M. in Treatise on Geochemistry *2nd edn* (2014).

[b32] Henderiks J. & Pagani M. Refining ancient carbon dioxide estimates: significance of coccolithophore cell size for alkenone-based pCO_2_ records. Paleoceanography 22, (2007).

[b33] van de Wal R. S., de Boer B., Lourens L. J., Köhler P. & Bintanja R. Reconstruction of a continuous high-resolution CO_2_ record over the past 20 million years. Clim. Past 7, 1459–1469 (2011).

[b34] Hopkinson B. M., Dupont C. L., Allen A. E. & Morel F. M. M. Efficiency of the CO_2_-concentrating mechanism of diatoms. Proc. Natl Acad. Sci. USA 108, 3830–3837 (2011). 21321195 10.1073/pnas.1018062108 PMC3054024

[b35] Laws E. A., Popp B. N., Cassar N. & Tanimoto J. ^13^C discrimination patterns in oceanic phytoplankton: likely influence of CO_2_ concentrating mechanisms, and implications for palaeoreconstructions. Funct. Plant Biol. 29, 323–333 (2002). 10.1071/PP01183 32689479

[b36] Hönisch B., Hemming N. G., Archer D., Siddall M. & McManus J. F. Atmospheric carbon dioxide concentration across the mid-Pleistocene transition. Science 324, 1551–1554 (2009). 19541994 10.1126/science.1171477

[b37] Zhang Y. G., Pagani M., Liu Z., Bohaty S. M. & DeConto R. A 40 million-year history of atmospheric CO_2_. Phil. Trans. R. Soc. A 371, 20130096 (2013). 24043869 10.1098/rsta.2013.0096

[b38] Lüthi D. *et al.* High-resolution carbon dioxide concentration record 650,000–800,000 years before present. Nature 453, 379–382 (2008). 18480821 10.1038/nature06949

[b39] Clark P. U. *et al.* The middle Pleistocene transition: characteristics, mechanisms, and implications for long-term changes in atmospheric *p*CO_2_. Quat. Sci. Rev. 25, 3150–3184 (2006).

[b40] Markovic S., Paytan A. & Wortmann U. G. Pleistocene sediment offloading and the global sulfur cycle. Biogeosciences 12, 3043–3060 (2015).

[b41] Tyrrell T. & Zeebe R. E. History of carbonate ion concentration over the last 100 million years. Geochim. Cosmochim. Acta 68, 3521–3530 (2004).

[b42] Beaufort L., Couapel M., Buchet N., Claustre H. & Goyet C. Calcite production by coccolithophores in the south east Pacific Ocean. Biogeosciences 5, 1101–1117 (2008).

[b43] Cubillos J. *et al.* Calcification morphotypes of the coccolithophorid *Emiliania huxleyi i*n the Southern Ocean: changes in 2001 to 2006 compared to historical data. Mar. Ecol. Prog. Ser. 348, 47–54 (2007).

[b44] Henderiks J. *et al.* Environmental controls on *Emiliania huxleyi* morphotypes in the Benguela coastal upwelling system (SE Atlantic). Mar. Ecol. Prog. Ser. 448, 51–66 (2012).

[b45] Poulton A. J., Young J. R., Bates N. R. & Balch W. M. Biometry of detached *Emiliania huxleyi* coccoliths along the Patagonian Shelf. Mar. Ecol. Prog. Ser. 443, 1–17 (2011).

[b46] Smith H. E. K. *et al.* Predominance of heavily calcified coccolithophores at low CaCO_3_ saturation during winter in the Bay of Biscay. Proc. Natl Acad. Sci. USA 109, 8845–8849 (2012). 22615387 10.1073/pnas.1117508109 PMC3384182

[b47] Poulton A. J. *et al.* Coccolithophores on the north-west European shelf: calcification rates and environmental controls. Biogeosciences 11, 3919–3940 (2014).

[b48] Ridgwell A. *et al.* From laboratory manipulations to Earth system models: scaling calcification impacts of ocean acidification. Biogeosciences 6, 2611–2623 (2009).

[b49] De Bodt C., Van Oostende N., Harlay J., Sabbe K. & Chou L. Individual and interacting effects of *p*CO_2_ and temperature on *Emiliania huxleyi* calcification: study of the calcite production, the coccolith morphology and the coccosphere size. Biogeosciences 7, 1401–1412 (2010).

[b50] Iglesias-Rodriguez M. D. *et al.* Phytoplankton calcification in a high-CO_2_ world. Science 320, 336–340 (2008). 18420926 10.1126/science.1154122

[b51] Langer G. & Bode M. CO_2_ mediation of adverse effects of seawater acidification in *Calcidiscus leptoporus*. Geochem. Geophys. Geosyst. 12, Q05001 (2011).

[b52] Henderiks J. & Pagani M. Coccolithophore cell size and the Paleogene decline in atmospheric CO_2_. Earth. Planet. Sci. Lett. 269, 576–584 (2008).

[b53] Foster G. L., Lear C. H. & Rae J. W. B. The evolution of *p*CO_2_, ice volume and climate during the Middle Miocene. Earth. Planet. Sci. Lett. 341-344, 243–254 (2012).

[b54] Taylor A. R., Chrachri A., Wheeler G., Goddard H. & Brownlee C. A voltage-gated H^+^ channel underlying pH homeostasis in calcifying coccolithophores. PLoS Biol. 9, e1001085 (2011). 21713028 10.1371/journal.pbio.1001085 PMC3119654

[b55] Langer G., Probert I., Nehrke G. & Ziveri P. The morphological response of *Emiliania huxleyi* to seawater carbonate chemistry changes: an inter-strain comparison. J. Nannoplankton Res. 32, 29–34 (2011).

[b56] Rickaby R. E. M., Henderiks J. & Young J. N. Perturbing phytoplankton: response and isotopic fractionation with changing carbonate chemistry in two coccolithophore species. Clim. Past. 6, 771–785 (2010).

[b57] Riebesell U. & Tortell P. D. in Ocean acidification eds Gattuso J. P., Hanson L. 99–121Oxford University Press (2011).

[b58] Young J., Poulton A. & Tyrrell T. Morphology of *Emiliania huxleyi* coccoliths on the North West European shelf-is there an influence of carbonate chemistry? Biogeosci. Discuss. 11, 4531–4561 (2014).

[b59] Suchéras-Marx B. & Henderiks J. Downsizing the pelagic carbonate factory: impacts of calcareous nannoplankton evolution on carbonate burial over the past 17 million years. Glob. Planet. Change. 123, 97–109 (2014).

[b60] John E. H. *et al.* Warm ocean processes and carbon cycling in the Eocene. Phil. Trans. R. Soc. A 371, 20130099 (2013). 24043871 10.1098/rsta.2013.0099

[b61] Kwon E. Y., Primeau F. & Sarmiento J. L. The impact of remineralization depth on the air–sea carbon balance. Nat. Geosci. 2, 630–635 (2009).

[b62] Keller M., Selvin R., Claus W. & Guillard R. Media for the culture of oceanic ultraphytoplankton. J. Phycol. 23, 633–638 (1987).

[b63] Bradshaw A., Brewer P., Shafer D. & Williams R. Measurements of total carbon dioxide and alkalinity by potentiometric titration in the GEOSECS program. Earth. Planet. Sci. Lett. 55, 99–115 (1981).

[b64] Brewer P., Bradshaw A. & Williams R. in The Changing Carbon Cycle: A Global Analysis eds Trabalka J. R., Reichle D. E. 348–370Springer (1986).

[b65] Popp B. N. *et al.* Effect of phytoplankton cell geometry on carbon isotopic fractionation. Geochim. Cosmochim. Acta 62, 69–77 (1998).

[b66] Menden-Deuer S. & Lessard E. J. Carbon to volume relationships for dinoflagellates, diatoms, and other protist plankton. Limnol. Oceanogr. 45, 569–579 (2000).

[b67] Montagnes D. J., Berges J. A., Harrison P. J. & Taylor F. Estimating carbon, nitrogen, protein, and chlorophyll a from volume in marine phytoplankton. Limnol. Oceanogr. 39, 1044–1060 (1994).

[b68] Zondervan I., Rost B. & Riebesell U. Effect of CO_2_ concentration on the PIC/POC ratio in the coccolithophore *Emiliania huxley*i grown under light-limiting conditions and different daylengths. J. Exp. Mar. Bio. Ecol. 272, 55–70 (2002).

[b69] Fuertes M. A., Flores J. A. & Sierro F. J. The use of circularly polarized light for biometry, identification and estimation of mass of coccoliths. Mar. Micropaleontol. 113, 44–55 (2014).

[b70] Beaufort L. Weight estimates of coccoliths using the optical properties (birefringence) of calcite. Micropaleontology 51, 289–297 (2005).

[b71] Beaufort L., Barbarin N. & Gally Y. Optical measurements to determine the thickness of calcite crystals and the mass of thin carbonate particles such as coccoliths. Nat. Protoc. 9, 633–642 (2014). 24556786 10.1038/nprot.2014.028

[b72] Flores J. A. *et al.* Sedimentation rates from calcareous nannofossil and planktonic foraminifera biostratigraphy in the Andaman Sea, northern Bay of Bengal, and Eastern Arabian Sea. Mar. Pet. Geol. 58, 425–437 (2014).

[b73] Shackleton N., Crowhurst S., Weedon G. & Laskar J. Astronomical calibration of Oligocene-Miocene time. Phil. Trans. R. Soc. London Ser. A 357, 1907–1929 (1999).

[b74] Flores J. & Sierro F. Revised technique for calculation of calcareous nannofossil accumulation rates. Micropaleontology 43, 321–324 (1997).

[b75] O'Dea S. A. *et al.* Coccolithophore calcification response to past ocean acidification and climate change. Nat. Commun. 5, 5363 (2014). 25399967 10.1038/ncomms6363 PMC4243242

[b76] Young J. in Calcareous Nannofossil Biostratigraphy 225–265 (1998).

[b77] Young J. & Ziveri P. Calculation of coccolith volume and its use in calibration of carbonate flux estimates. Deep Sea Res. Part II 47, 1679–1700 (2000).

[b78] Hoffmann R. *et al.* Insight into *Emiliania huxleyi* coccospheres by focused ion beam sectioning. Biogeosciences 12, 825–834 (2015).

[b79] Gibbs S. J. *et al.* Species-specific growth response of coccolithophores to Palaeocene-Eocene environmental change. Nat. Geosci. 6, 218–222 (2013).

[b80] Farmer E. C., Kaplan A., de Menocal P. B. & Lynch-Stieglitz J. Corroborating ecological depth preferences of planktonic foraminifera in the tropical Atlantic with the stable oxygen isotope ratios of core top specimens. Paleoceanography 22, (2007).

[b81] Spero H. J., Mielke K. M., Kalve E. M., Lea D. W. & Pak D. K. Multispecies approach to reconstructing eastern equatorial Pacific thermocline hydrography during the past 360 kyr. Paleoceanography 18, (2003).

[b82] Tedesco K., Thunell R., Astor Y. & Muller-Karger F. The oxygen isotope composition of planktonic foraminifera from the Cariaco Basin, Venezuela: Seasonal and interannual variations. Mar. Micropaleontol. 62, 180–193 (2007).

[b83] Diester-Haass L. *et al.* Mid-Miocene paleoproductivity in the Atlantic Ocean and implications for the global carbon cycle. Paleoceanography 24, PA1209 (2009).

[b84] Pagani M., Zachos J. C., Freeman K. H., Tipple B. & Bohaty S. Marked decline in atmospheric carbon dioxide concentrations during the Paleogene. Science 309, 600–603 (2005). 15961630 10.1126/science.1110063

[b85] Pagani M., Arthur M. A. & Freeman K. H. Miocene evolution of atmospheric carbon dioxide. Paleoceanography 14, 273–292 (1999).

[b86] Zachos J. C., Dickens G. R. & Zeebe R. E. An early Cenozoic perspective on greenhouse warming and carbon-cycle dynamics. Nature 451, 279–283 (2008). 18202643 10.1038/nature06588

