# Supplementary material for: Decrease in coccolithophore calcification and CO2 since the middle Miocene
Source: Nat Commun. 2016 Jan 14;7:10284. doi: 10.1038/ncomms10284 (PMC4735581; doi:10.1038/ncomms10284)
Supplement: Supplementary Figures and Supplementary Tables — Supplementary Figures 1-13 and Supplementary Tables 1-3, Supplementary Methods, Supplementary References [file ncomms10284-s1.pdf]

Supplementary Figures for "Decrease in coccolithophore calcification and CO_2 since the middle Miocene" by Clara T. Bolton *et al.*

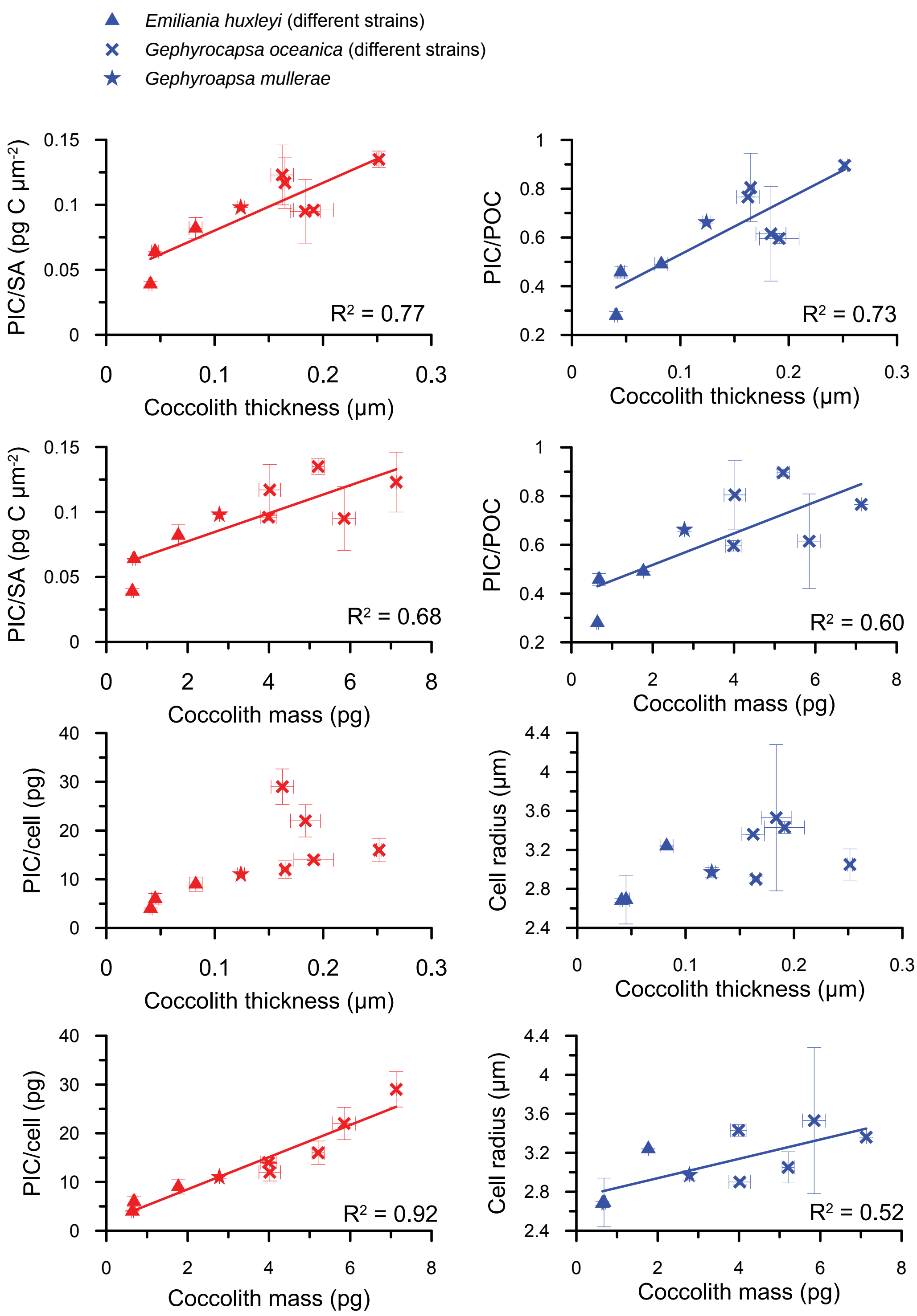

Supplementary Figure 1: Results from culture experiments with eight different strains of modern Noëlaerhabdaceae. Symbols denote the average for each experiment, and lines denote the range between replicate bottles for the experiment.

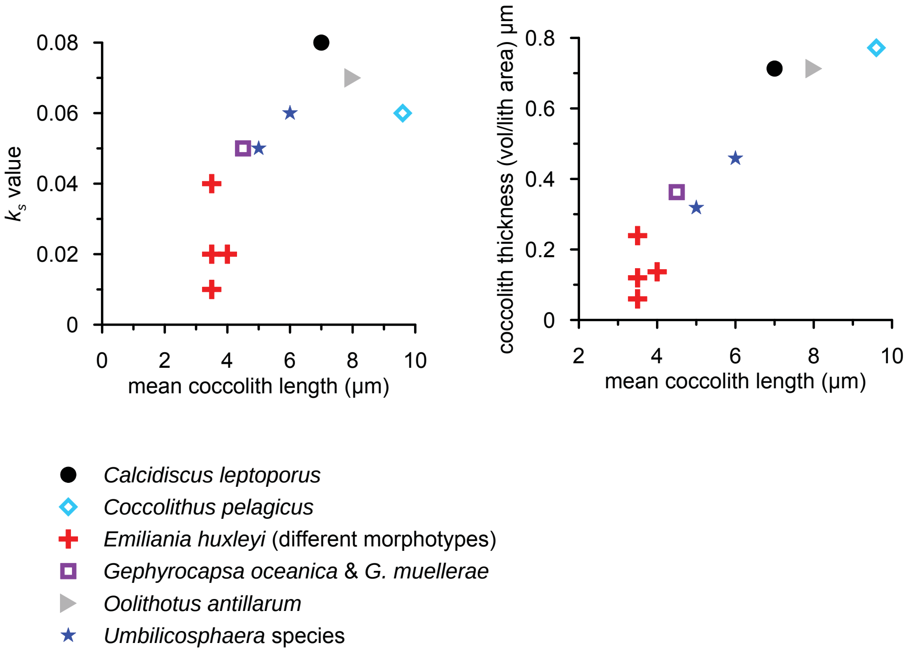

Supplementary Figure 2: k_s and thickness values for modern placolith (*Coccolithus*, *Calcidiscus*, *Emiliania*, *Gephyrocapsa*, *Umbilicosphaera*, *Oolithus*) coccoliths plotted versus mean coccolith length. k_s data are from ref. 1. Thickness was calculated using k_s values and mean lengths from ref. 1. Coccolith widths, also required to calculate thickness, were estimated using published estimates of circularity (ref. 2) and estimates of *E. huxleyi* and *Gephyrocapsa oceanica* circularity from our culture experiments reported in this paper.

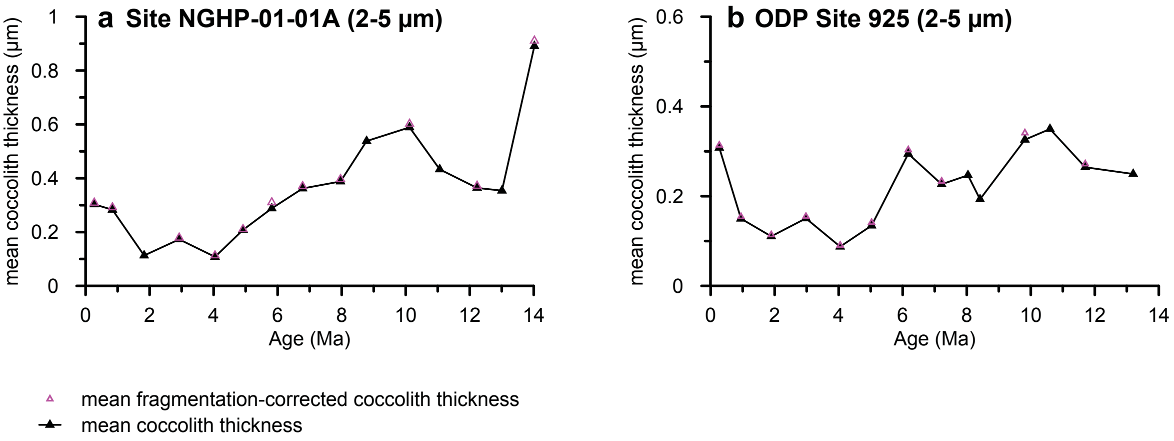

Supplementary Figure 3: Mean coccolith thickness for all coccoliths 2-5 µm, at **a**: Site NGHP-01-01A, and **b**: ODP Site 925. Thickness (black symbols, line) and fragmentation-corrected thickness (pink symbols) are shown.

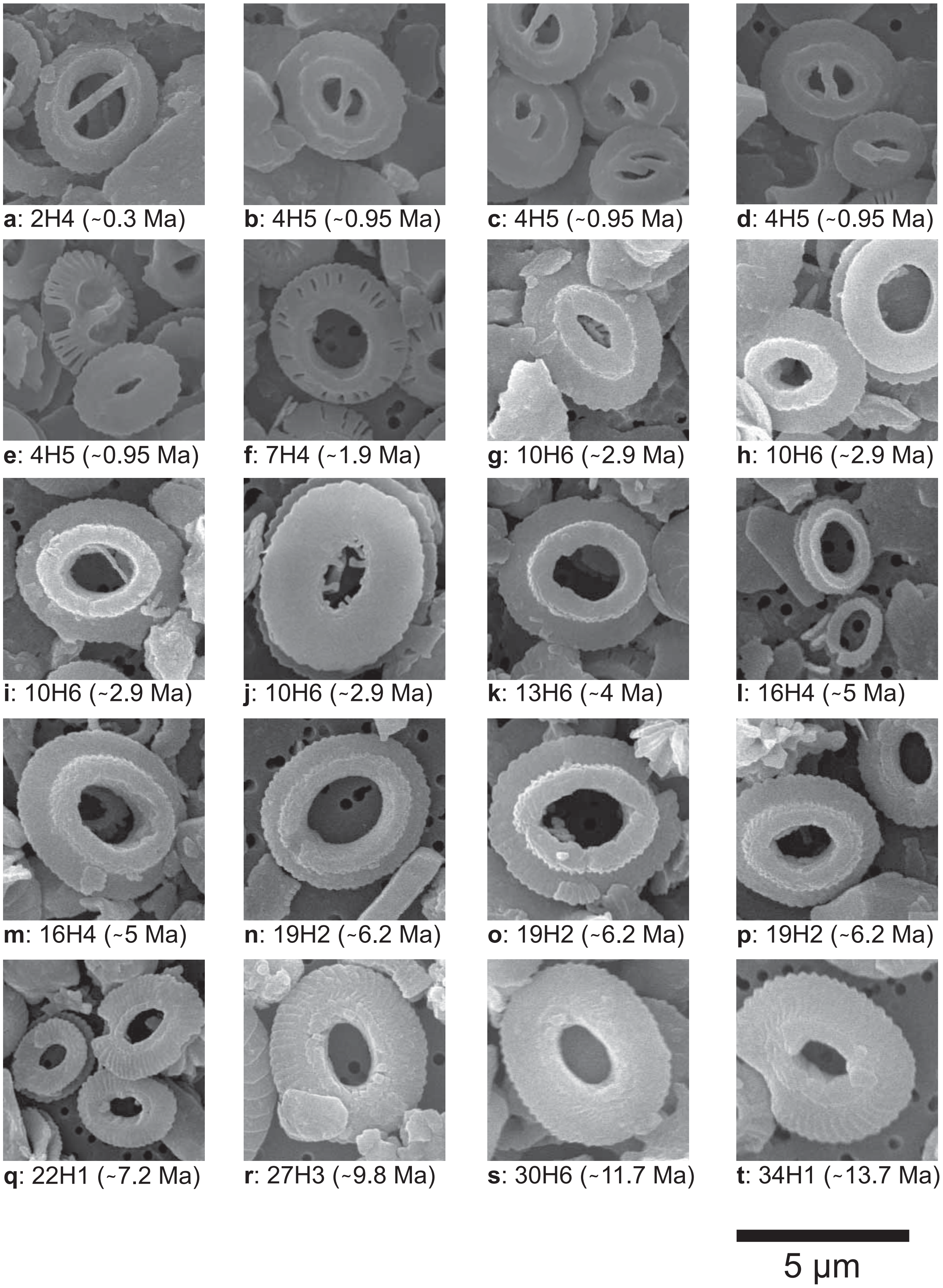

Supplementary Figure 4: SEM plate illustrating the variety of Noëlaerhabdaceae coccolith morphology found at ODP Site 925.

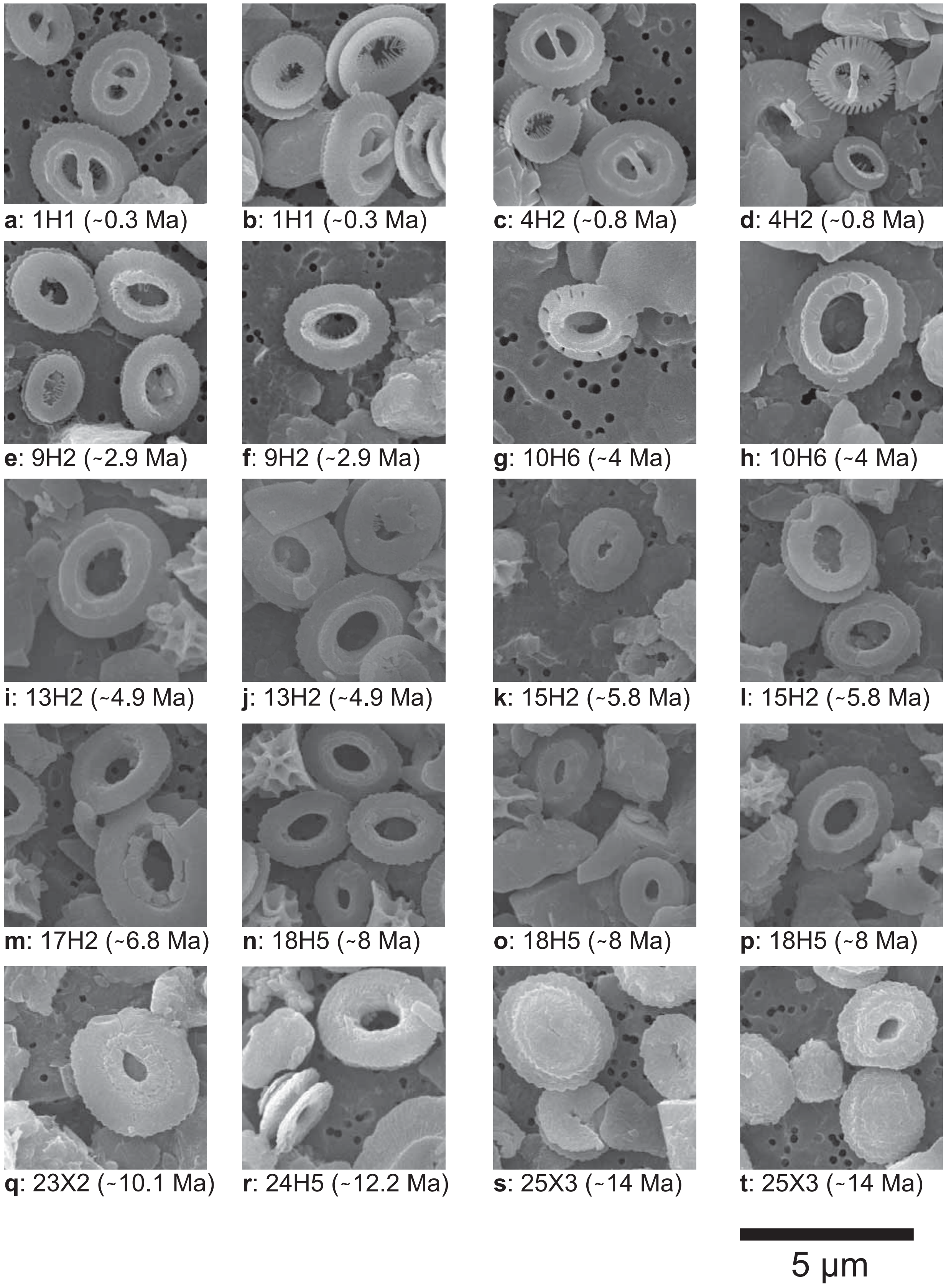

Supplementary Figure 5: SEM plate illustrating the variety of Noëlaerhabdaceae coccolith morphology found at Site NGHP-01-01A.

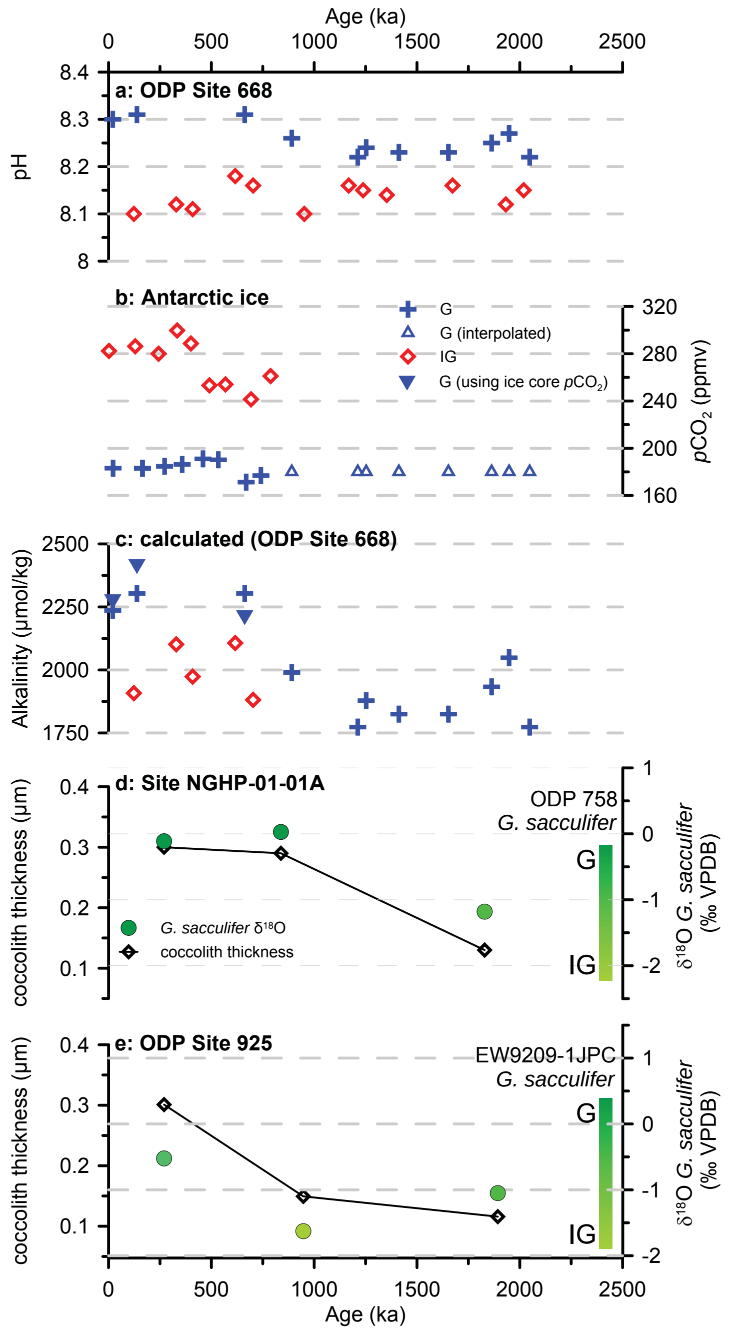

Supplementary Figure 6: **a**: pH estimates for glacial and interglacial endmembers[3], **b**: pCO_2 estimates for glacial and interglacial end-members (compiled in ref. 4) and the assumption of constant glacial pCO_2 prior to the ice core record, **c**: estimated surface alkalinity at ODP Site 668 in the tropical Atlantic based on these two parameters, following calculations described in the Methods section, **d**: size-normalised coccolith thickness (2-5 μm) and *G. sacculifer* $\delta^{18}O$ data at Site NGHP 01-01A, **e**: size-normalised coccolith thickness (2-5 μm) and *G. sacculifer* $\delta^{18}O$ data at Site 925. In **d** and **e**, the green bar on the right shows the last glacial (G) to current interglacial (IG) range in *G. sacculifer* $\delta^{18}O$ for nearby sites where high-resolution data are available: equatorial Indian Ocean data from ODP Site 758 (ref. 5) and western equatorial Atlantic data from Site EW9209-1JPC (ref. 6).

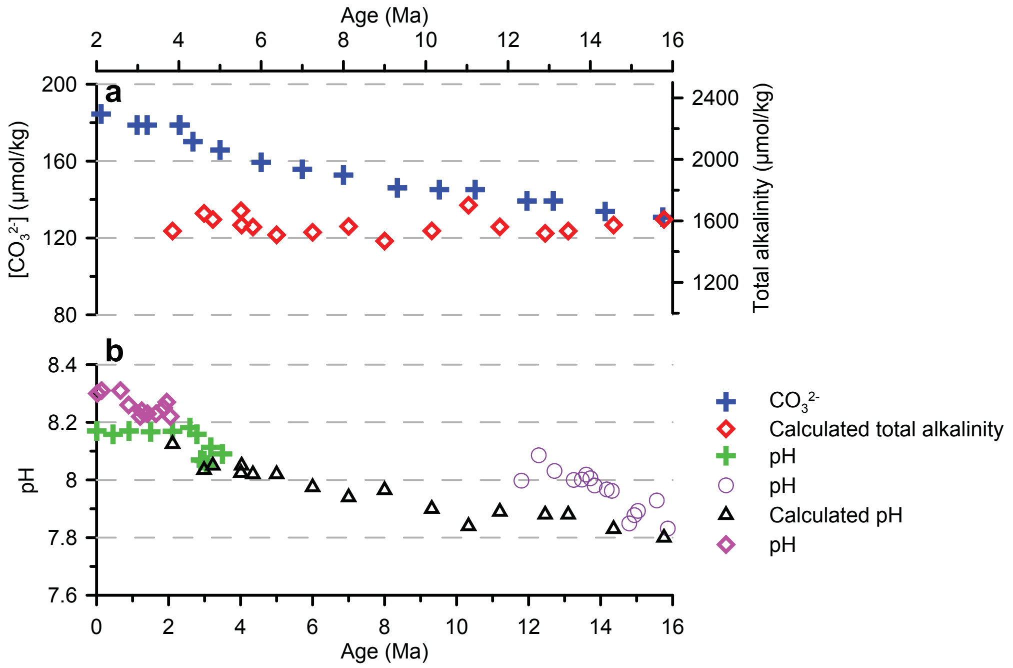

Supplementary Figure 7: **a**: Long-term changes (16-2 Ma) in carbonate ion concentration (ref. 7) and alkalinity (calculated from [CO_3^{2-}] (ref. 7) and [CO_{2aq}] determined in this study as described in the Methods section), **b**: measured (boron isotopes) and calculated (using [CO_3^{2-}] (ref. 7) and [CO_{2aq}]) pH changes over the interval 16-0 Ma (references 8-10).

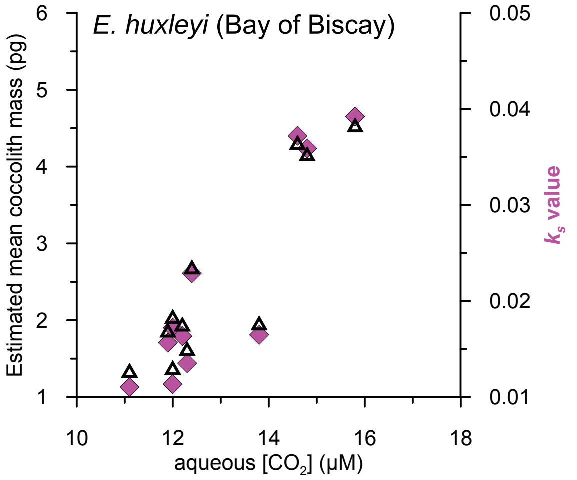

Supplementary Figure 8: Calculated *E. huxleyi* coccolith mass versus [CO_{2aq}]. Original data (carbonate chemistry and % overcalcified *E. huxleyi*) from reference 11, in which water samples from the Bay of Biscay were analysed monthly between September 2008 and August 2009. Here, we estimate mean coccolith mass using the percentage of overcalcified *E. huxleyi* in each sample (ref. 11) and published mean coccolith mass values for *E. huxleyi* Type A overcalcified (4.6 pg) and *E. huxleyi* Type A undercalcified (1.2 pg)[1].

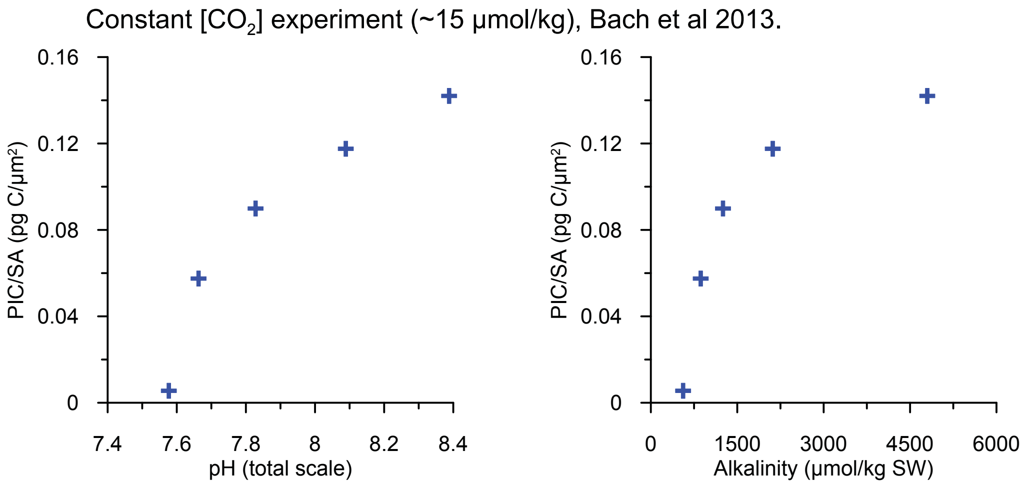

Supplementary Figure 9: Results from a constant [CO_2] experiment with *Emiliania huxleyi*[12], showing the increase in calcification (illustrated as cellular PIC per cell surface area calculated from POC) with increasing pH and increasing alkalinity.

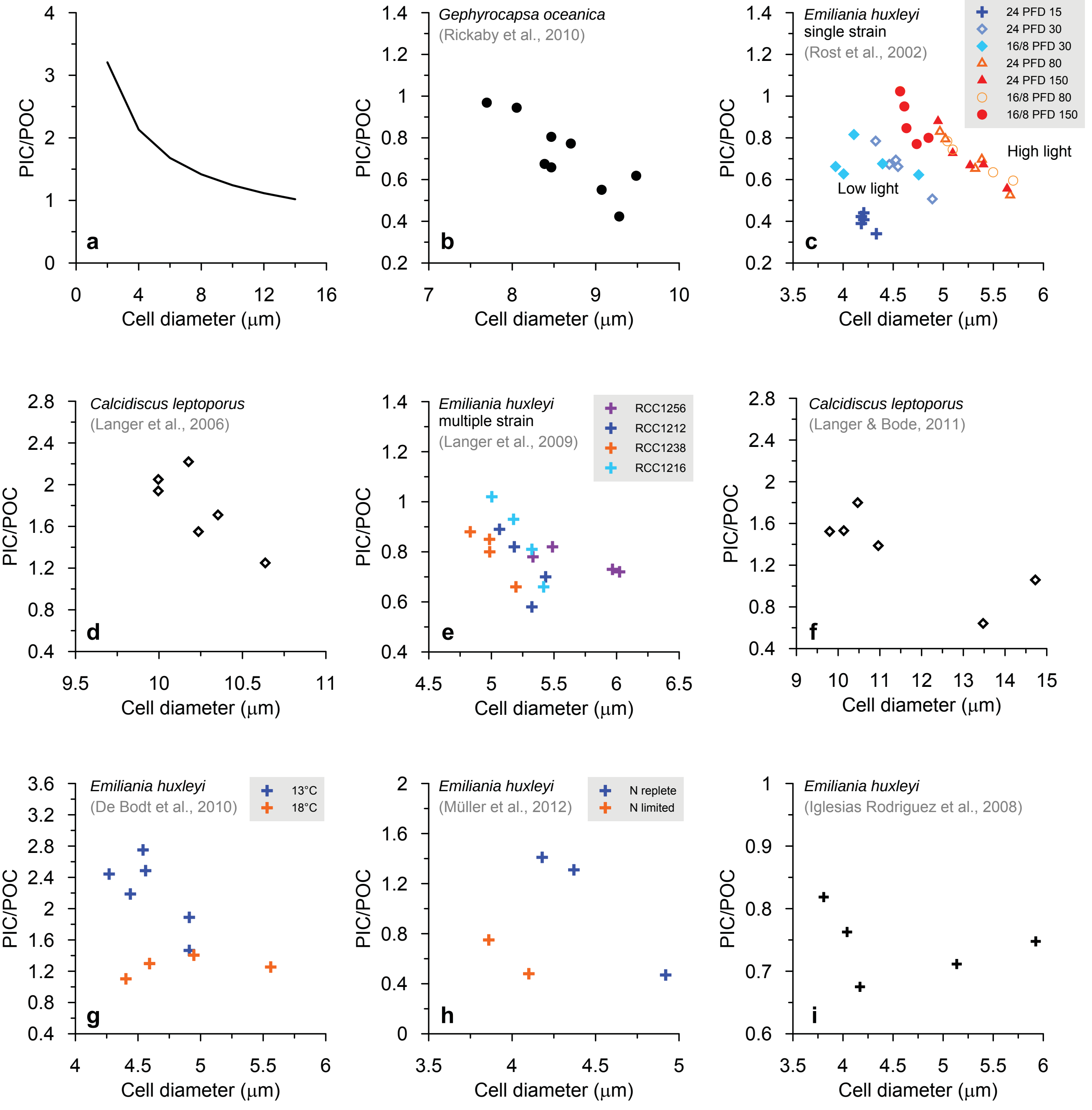

Supplementary Figure 10: Coccolithophore PIC to POC ratios versus cell size in culture. **a**: The theoretical relationship between PIC to POC ratio and cell size assuming constant coccolith thickness. **b** to **i**: PIC/POC ratios vs cell diameter from published culture experiments with different species and strains (note different scales). All cell diameters (except **h** where cell size was measured by the authors) were calculated from POC per cell measurements in the experiments, using the well-constrained relationship between cell size and POC from ref. 13. Most experiments show a decrease in PIC to POC with increasing cell size, as predicted in **a**. Data are from refs. 14 to 21.
Not all studies indicate timing of sampling relative to the cell division cycle.

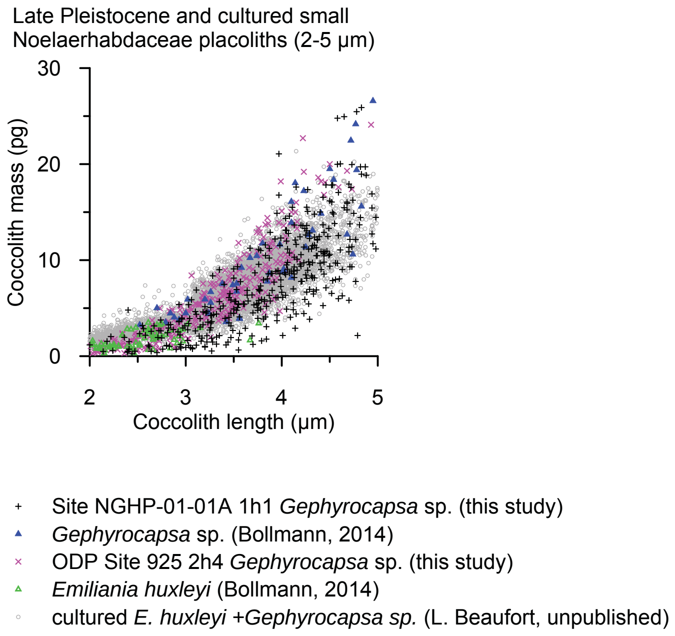

Supplementary Figure 11: A comparison of coccolith mass data derived from three recently published variations on the birefringence method. We include our data from ~0.3 Ma samples at ODP Site 925 (pink crosses) and Site NGHP-01-01A (black crosses), which are both exclusively composed of *Gephyrocapsa* coccoliths and measured following ref. 22. Also plotted are Holocene coccolith mass data for *Gephyrocapsa mullerae* and *G. oceanica* (blue triangles) and *E. huxleyi* (green triangles), published in ref. 23. Additionally, data for mixed *Gephyrocapsa* sp. and *E. huxleyi* coccoliths grown in culture and measured according to ref. 24 are shown (grey circles; L. Beaufort, unpublished data, 2014). Whereas the methods of ref. 22 and ref. 23 photograph coccoliths using circular polarised light, in ref. 24 coccoliths are imaged under cross-polarised light at three specific orientations and create a composite image so as to eliminate the extinction cross. Taking into account the expected variability in mass for coccoliths of a given size resulting from variable geographical locations, ages and growth conditions in the datasets we compare, there is very good agreement between Noëlaerhabdaceae coccolith mass values for the three birefringence-based methods. Calibrations applied to convert grey level to thickness also differ slightly between methods. A sensitivity test performed on our data to account for the sigmoidal shape of the grey level-thickness relationship in the newest published calibrations showed an increase in absolute thickness values, particularly for the smallest coccoliths, but no change in the temporal thickness trends at either site meaning that our interpretations are not affected.

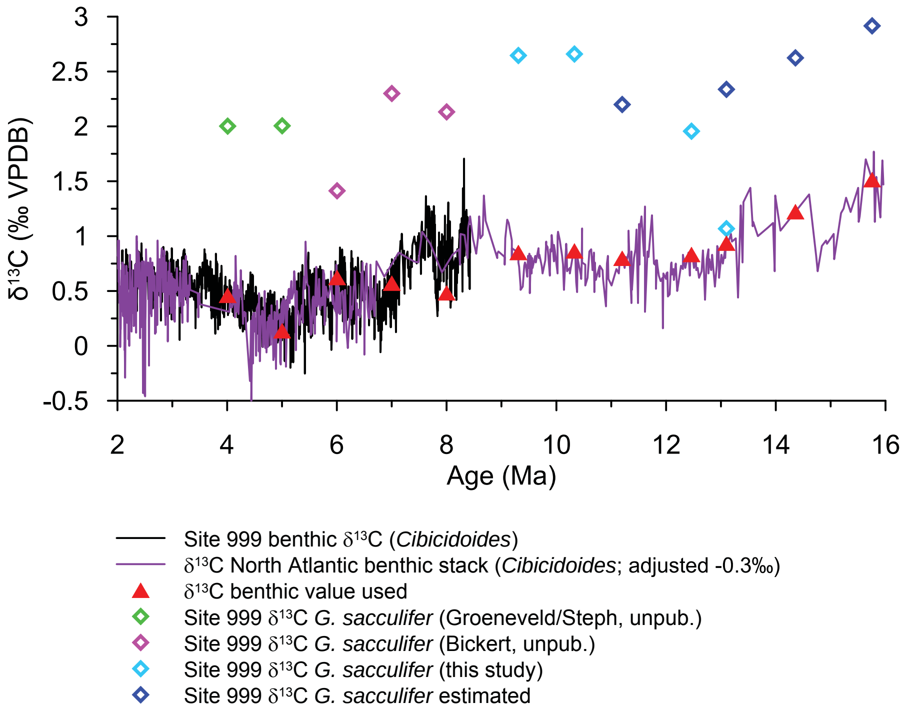

Supplementary Figure 12: Benthic and planktic foraminiferal $\delta^{13}C$ data from ODP Site 999. *G. sacculifer* data are from Groeneveld & Steph (unpublished), Bickert (unpublished) and this study (Supplementary Data 2). The ODP Site 999 benthic record (Black line, refs 25, 26) was extended using a North Atlantic compilation (Purple line, ref. 27) corrected for an offset of -0.3‰. Diamonds show *G. sacculifer* values used to estimate $\delta^{13}C_{DIC}$.

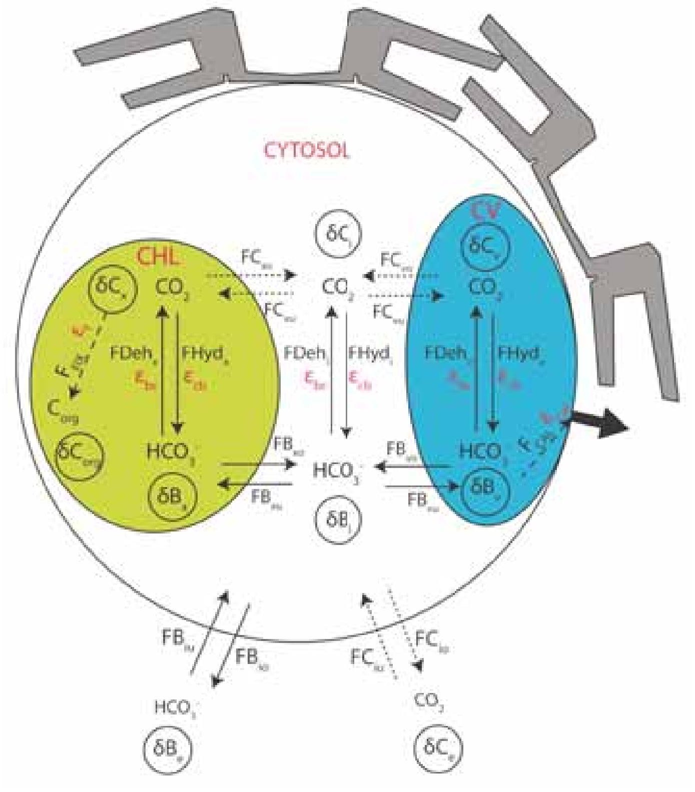

Supplementary Figure 13:
(diagram modified from reference 28). Illustration of mass balance of carbon, and isotopic composition in coccolithophore cell keyed to the relevant mass balance equations using the ACTI-CO model, as described in reference 28. Fractionation factors are identical to reference 28. CHL indicates chloroplast and CV is coccolith vesicle. Labelling of fluxes and isotopic compositions follows the convention of reference 29 with the addition of the subscript v to denote fluxes within and to the CV, such that e, i, x, and v denote respectively external, intracellular (cytosol), chloroplast, and CV, and u and o refer to uptake and outflux, respectively. Notation is as in reference 29.

(1) Chloroplast CO_2 :

$$0 = \delta C_i FC_{xu} + (\delta B_x + \varepsilon_{bc}) FDeh_x - \delta C_x FC_{xo} - (\delta C_x + \varepsilon_{cb}) FHyd_x - (\delta C_x + \varepsilon_f) F_{FIX}$$

(2) Chloroplast HCO_3^- :

$$0 = \delta B_i FB_{xu} + (\delta C_x + \varepsilon_{cb}) FHyd_x - \delta B_x FB_{xo} - (\delta B_x + \varepsilon_{bc}) FDeh_x$$

(3) Coccolith vesicle CO_2 :

$$0 = \delta C_i FC_{vu} + (\delta B_v + \varepsilon_{bc}) FDeh_v - \delta C_v FC_{vo} - (\delta C_v + \varepsilon_{cb}) FHyd_v$$

(4) Coccolith vesicle HCO_3^- :

$$0 = \delta B_i FB_{vu} + (\delta C_v + \varepsilon_{cb}) FHyd_v - \delta B_v FB_{vo} - (\delta B_v + \varepsilon_{bc}) FDeh_v - (\delta B_v + \varepsilon_{cal}) F_{CAL}$$

(5) Cytosol CO_2 :

$$0 = \delta C_e FC_{iu} + \delta C_x FC_{xo} + \delta C_v FC_{vo} + (\delta B_i + \varepsilon_{bc}) FDeh_i - \delta C_i (FC_{io} + FC_{xu} + FC_{vu}) - (\delta C_i + \varepsilon_{cb}) FHyd_i$$

(6) Cytosol HCO_3^- :

$$0 = \delta B_e FB_{iu} + \delta B_x FB_{xo} + \delta B_v FB_{vo} + (\delta C_i + \varepsilon_{cb}) FHyd_i - \delta B_i (FB_{io} + FB_{xu} + FB_{vu}) - (\delta B_i + \varepsilon_{bc}) FDeh_i$$

Supplementary Table 1: Coccolithophore strains cultured for this study

RCC	Strain	Genus	Species	Isolation	Lat.	Long.
1257	AC447, BP81	*Emiliania*	*huxleyi*	Probert I. (1/7/1999)	+63° 27'	-20° 14'
1292	AC638, PR3F1	*Gephyrocapsa*	*oceanica*	Probert I. (20/3/2003)	+14° 49'	-67° 3'
1803	DM2-3	*Gephyrocapsa*	*oceanica*	Probert I. (9/12/2008)	+4° 7'	+118° 39'
3370	CHC184	*Gephyrocapsa*	*mullerae*	P. Von Dassow (1/11/2011)	-30° 15'	-71° 42'
3651	VFEh1	*Emiliania*	*huxleyi*	Probert I. (20/9/2012)	+43° 41'	+7° 19'
3724	NG5	*Gephyrocapsa*	*oceanica*	K. Hagino (25/10/2011)	+32° 25'	+128° 40'
3728	NG11	*Gephyrocapsa*	*oceanica*	K. Hagino (25/10/2011)	+32° 25'	+128° 40'
4003	CHC310	*Emiliania*	*huxleyi*	P. von Dassow (1/10/2011)	-33° 38'	-78° 49'

Supplementary Table 2: Correlation between coccolith mass and thickness and cell size and calcification parameters

**Correlation**	Coccolith thickness	Coccolith mass	PIC/SA	Cell radius	PIC/POC	PIC/cell
Coccolith thickness						
Coccolith mass	0.83 (0.0060)					
PIC/SA	**0.88 (0.0020)**	0.83 (0.0059)				
Radius	0.59 (0.0930)	**0.73 (0.0270)**	0.48			
Strain avg PIC/POC	**0.86 (0.0033)**	0.77 (0.0150)	0.99	0.34		
PIC/cell	0.66 (0.0540)	**0.96 (4.2E-05)**	0.71	0.76 (.017)	**0.64**	

Values in brackets represent the significance level (p-value) of the regression.

Supplementary Table 3: Notation used in ACTI-CO numerical model (ref. 28) and Supplementary Figure 13. All fluxes are given in units of 10^{-17} mol s^{-1}.

Symbol	Definition	Default value	Parameterization employed where differing from ref. 28	Units
FC_{iu}	CO_2 flux from extracellular media to cell cytosol		Set from permeability, membrane surface area, and CO_2 concentration as detailed in reference 28	
FC_{xo}	CO_2 flux from chloroplast to cytosol			
FC_{xu}	CO_2 flux from cytosol to chloroplast			
FC_{io}	CO_2 flux from cell cytosol to extracellular media			
FC_{vu}	CO_2 flux from cytosol to coccolith vesicle			
FC_{vo}	CO_2 flux from coccolith vesicle to cytosol	0		
FB_{iu}	HCO_3^- flux from extracellular media into cell cytosol		0.5*(FB_{vu} + FB_{xu})	
FB_{xu}	HCO_3^- flux from cytosol to chloroplast		As illustrated in Figure 8	
F_{FIX}	Photosynthetic carbon fixation flux (μ*POC/cell)		μ=(0.4)<br>POC/cell estimated from biovolume and cell diameter estimated from coccolith length	
F_{CAL}	Calcification flux (μm*PIC/cell)		μ=(0.4)<br>PIC/cell derived from PIC/POC of 0.6	
FB_{vu}	HCO_3- flux from cytosol to coccolith vesicle		Set to solve $\varepsilon_{calcite}$	
f_{cv}	Ratio of effective permeability of coccolith vesicle to that of plasma membrane		0.4	
f_{chl}	Ratio of effective permeability of chloroplast to that of plasma membrane		0.3	
FB_{xo}	HCO_3^- flux from chloroplast to cytosol	0		
FB_{io}	HCO_3^- flux from cell cytosol to extracellular media	0		
$FDeh_i$	dehydration of HCO_3^- to CO_2 in cell cytosol		As described in reference 28	
$FHyd_i$	hydration of CO_2 to HCO_3^- in cell cytosol			
$FHyd_x$	hydration of CO_2 to HCO_3^- in chloroplast			
$FDeh_x$	dehydration of HCO_3^- to CO_2 in chloroplast			
$FHyd_v$	hydration of CO_2 to HCO_3^- in coccolith vesicle			
$FDeh_v$	dehydration of HCO_3^- to CO_2 in coccolith vesicle			
FB_{vo}	HCO_3^- flux from coccolith vesicle to cytosol	0		
δC_e	$\delta^{13}C$ of CO_2 in external media			‰
δC_i	$\delta^{13}C$ of CO_2 in cell cytosol			‰
δC_x	$\delta^{13}C$ of CO_2 in chloroplast			‰
δB_e	$\delta^{13}C$ HCO_3^- in external media			‰
δB_i	$\delta^{13}C$ HCO_3- in cell cytosol			‰
δB_x	$\delta^{13}C$ HCO_3- in chloroplast			‰
δC_v	$\delta^{13}C$ of CO_2 in coccolith vesicle			‰
δB_v	$\delta^{13}C$ of HCO_3- in coccolith vesicle			‰
δC_{org}	$\delta^{13}C$ of organic matter			‰

Supplementary Methods

Carbon isotopes in alkenones and ε_p and [CO_{2aq}] calculations

Lipids were extracted from sediments and alkenones subsequently isolated as described in ref. 8. Gas chromatography (GC) was performed with an Agilent 7890A chromatograph equipped with a flame ionisation detector and an Agilent J&W HP-1 fused silica column (19091Z-015, 50 m x 0.32 mm internal diameter) coated with CP Sil5-CB stationary phase (dimethylpolysiloxane equivalent, 0.12 µm film thickness). The oven temperature was programmed from 70 to 130 °C at 20 °C min^{-1}, to 300 °C (held 25 min) at 4 °C and H_2 was used as carrier gas. Compounds were quantified using a C_{36}*n*-alkane that had been added to the alkenone sub-fraction prior to injection. Compound-specific isotope analyses were performed using a GC-combustion-isotope ratio mass spectrometer (GC–C–IRMS) with an Agilent 7890A GC coupled to a Nu Instruments Perspective IRMS. The GC column and temperature programme were as for GC analysis. The internal standard added to the alkenone sub-fraction was of known isotopic composition to ensure instrument stability[8]. Isotope ratio values are reported as δ values ($\delta^{13}C$, ‰) and reproducibility was better than 0.7 ‰.

$\varepsilon_{p37:2}$ values (hereafter ε_p) were calculated from $\delta^{13}C$ values of di-unsaturated alkenones and calcite tests of planktic foraminifers (*G. sacculifer*) (Supplementary Data 2) measured in samples from ODP Site 999 (12°44' N, 78°44' W; water depth 2830 m, Fig. 1) to extend a previously published record[8]. ε_p trends are predominantly driven by changes in alkenone $\delta^{13}C$.

ε_p was calculated using the equation:

$$\varepsilon_{p37:2} = [(\delta CO_{2(aq)} + 1000) / (\delta^{13}C_{haptophyte\ biomass} + 1000)-1]*1000 \quad (1)$$

where $\delta CO_{2(aq)}$ is calculated from the $\delta^{13}C$ of the foraminifer *G. sacculifer*, the temperature-dependent relationship between $\delta CO_{2(aq)}$ and $\delta CaCO_3$ (see ref. 8 and refs therein) and estimated mixed layer temperatures. For samples at 4 and 5 Ma, *G. sacculifer* data are from J. Groeneveld & S. Steph (unpublished). From 6 Ma to 8 Ma, *G. sacculifer* data are from T. Bickert (unpublished). All other *G. sacculifer* $\delta^{13}C$ values were generated for this study (as described above for the other sites) (Supplementary Data 2). For three samples in the older part of the record, *G. sacculifer* could not be extracted in sufficient numbers for reliable isotopic analysis, hence we estimated *G. sacculifer* $\delta^{13}C$ from benthic foraminiferal $\delta^{13}C$, using the average $\delta^{13}C$ gradient between *Cibicidoides sp.* and *G. sacculifer* at ODP Site 999 (1.4 ‰). Benthic foraminiferal $\delta^{13}C$ (*Cib.)* for ODP Site 999 extends back to 8.4 Ma[25,26] and was extended to older periods using a compilation of North Atlantic sites (ODP/DSDP Sites 553, 558, 563, 607, 608, and 959) taken from ref. 27 (Supplementary Fig. 12). The North Atlantic compilation was adjusted by -0.3‰ to account for the mean offset relative to ODP Site 999 benthic $\delta^{13}C$ during the interval where the two records overlap (2-8.4 Ma) (Supplementary Fig. 12). For one sample corresponding to 13.1 Ma, planktic foraminferal $\delta^{13}C$ shows no gradient compared to benthic $\delta^{13}C$. Since there is no independent evidence for homogenization of the water column at this time (e.g. in coccolith assemblages), we infer that planktic foraminifers may be altered by recrystallization in the deep-water or sediment environment. Hence for this sample, we also use a value for *G. sacculifer* $\delta^{13}C$ calculated assuming a planktic-benthic gradient of 1.4 ‰.

Given the absence of tri-unsaturated alkenones in sediments samples, it was not possible to estimate sea surface temperatures (SSTs) from the U^{k}_{37}' index, and therefore maximum and minimum SST estimates for ODP Site 999[28] were used in ε_p calculations to calculate $\delta^{13}C_{CO2(aq)}$[8].

Propagated analytical uncertainty of ε_p for maximum and minimum SST scenarios (error bars in Figure 7a) = $\sqrt{((1 \text{ SD } \delta^{13}C_{\text{haptophyte biomass}}{}^2)+(0.05^2))}$. (2)

Calculations of aqueous CO_2 concentrations from ε_p and the various approaches we use to constrain temporal variation in b, thus isolating the component of variation in ε_p driven by $[CO_{2aq}]$, are described in the main text. The absolute values of b and $[CO_{2aq}]$ are much more poorly constrained than the trends, imparting a greater uncertainty in the absolute values of $[CO_{2aq}]$ and atmospheric pCO_2 than in the temporal trend. To correct for cell size changes, given that the trend in Noëlaerhabdaceae coccolith size observed in our sites is similar to that found in other tropical sites[30,31], we use our record of the mean length of coccoliths from the ODP Site 925 and NGHP-01-01A records because these precise determinations incorporate all the suspected calcifying alkenone-producing taxa (all Noëlaerhabdaceae), unlike the published record for the time interval from ODP Site 999[30]. To calculate the effect of size change on b, we apply the correction approach suggested by refs 32,33 using a reference b value = 150, to the size record of ODP 925 and NGHP-01-01A. This record of b from each site is then linearly interpolated to the sample ages of ε_p determinations at Site 999. The single exception is the 9.31 Ma point for ODP 925 because the adjacent ages (9.82 Ma, 8.42 Ma) straddle the major transition in cell size, whereas the 8.78 Ma sample in NGHP-01-01A precedes the size transition, suggesting that at 9.31 Ma large sizes prevailed. Therefore for 9.31 Ma at ODP 925, we estimate a b value 0.95 * the b value at 10.3 Ma - the scaling observed in b between these ages at Site NGHP-01-01A. With our choice of reference b = 150, the size-scaled b values range from 164 to 89 for NGHP-01-01A and 74 to 149 for ODP 925. As our focus is on ascertaining the trends, rather than absolute values, of b, we compensate for the difference in absolute values by adding 8.45 (the average offset between the two sites over the whole time interval) to the b estimate of ODP 925. The deviations in trend between the two sites are shown by the maximum and minimum b estimates in Figure 7d (error bars). This formulation describes the influence of cell size on ε_p assuming that changing cell size does not entail a change in maximum growth rate. Its use is consistent with the observation that for coccolithophores, the size dependence of growth rates is very small, equivalent to less than a 5 % increase in growth rate for the observed reduction in cell diameter from 4 to 2.7 μm, based on a comparison of coccolithophore growth rates in multiple culture studies[34].

The potential effect of changing growth rates on ε_p and calculated $[CO_{2aq}]$ is evaluated via changes in the b value, following previous studies[8]. As indicators of potential variations in growth rates, we examine variations in coccolith Sr/Ca ratios suggested to correlate positively with growth rate and the "b" physiological coefficient[35], and alkenone accumulation rates in sediment that may serve as a proxy for productivity of alkenone producers, particularly in situations where preservation potential is stable[36]. Given stable seawater Sr ratios over this time period[37], coccolith Sr/Ca might be expected to track growth rates of alkenone producers. We use Sr/Ca data from ODP Site 999, from the size fraction dominated by coccoliths from the alkenone-producing Noëlaerhabdaceae family[28]. Highest coccolith Sr/Ca occurs from 13-10 Ma, coinciding with a local maximum in alkenone mass accumulation rates (Fig. 7c), although the magnitude of Sr/Ca change in this interval may be amplified somewhat by a higher relative contribution of detrital Sr due to the low $CaCO_3$ content of sediments during the "carbonate crash". In addition, a peak in alkenone accumulation rate occurs at 8 Ma. The higher resolution alkenone mass accumulation rate data for the last 5 Ma[8] evidences higher

frequency variability. From these two indicators, we normalised the variation in each indicator and averaged these normalised variations to establish a composite growth rate curve for the interval 16-6 Ma to estimate temporal variation in b (Fig. 7d). There is as yet no calibration of the magnitude of growth rate change implied by either indicator; we use an amplitude of 25, consistent with that explored in ref. 8 and which is within the large uncertainty in the slope of relationship between Sr/Ca and b value in the Equatorial Pacific[35]. To illustrate the sensitivity of b to this choice, we also show growth rate variations with amplitude 20 % lower and higher (i.e., range of b of 20 or 30; error bars in Figure 7d). For the period studied in ref. 8, where ε_p data are not accompanied by Sr/Ca measurements, we do not simulate variations in growth rate. Adding this estimated variation in b to that inferred to result from cell size changes results in a range of b of 87 to 167 (Fig. 7d). Choice of an alternate reference b value produces very similar temporal trends of the reconstructed [CO_{2aq}] record but different absolute values; higher b reference values of 190 yields a decline in [CO_{2aq}] from 16 to 8 μM; assumption of lower b reference values of 138 yields a decline in [CO_{2aq}] from 11 to 6 μM.

Maximum and minimum [CO_{2aq}] estimates, calculated using data from Site 999 (this study and ref. 8), are shown as shading in Figure 7e and include uncertainty in ε_p as illustrated in Figure 7a, as well as uncertainty in estimates of b, as illustrated in Figure 7d. For size corrected data, maximum [CO_{2aq}] was calculated using the upper limit of ε_p (calculated using SST max, *G. sacculifer* $\delta^{13}C$ +1SD, and $\delta^{13}C_{haptophyte\ biomass}$ -1SD; see error bars in Fig. 7a), and the upper limit of b; whereas minimum [CO_{2aq}] was calculated using the lower limit of ε_p (calculated using SST min, *G. sacculifer* $\delta^{13}C$ -1SD, and $\delta^{13}C_{haptophyte\ biomass}$ +1SD, see error bars in Fig. 7a) and the lower limit of b (orange shading in Fig. 7e). For the size and growth-rate corrected data, the maximum and minimum [CO_{2aq}] were calculated analogously but using the b, which incorporates both size and growth rate terms (pink shading in Fig. 7e).

The effect of changing active uptake on [CO_{2aq}] estimates

To illustrate the potential impact of changes in active uptake of carbon on ε_p and calculated [CO_{2aq}], we use the ACTI-CO cell model of carbon fluxes in coccolithophores[28] (Supplementary Figure 13 and Supplementary Table 3). We impose several possible dependencies of active HCO_3^- transport to the chloroplast as a function of [CO_{2aq}], and for each, solve for the [CO_{2aq}] required to match observed ε_p at ODP 999. Laboratory culture experiments suggest that active HCO_3^- transport to the chloroplast becomes more significant at low [CO_{2aq}], with the ratio of chloroplast HCO_3^- transport to diffusive CO_2 uptake following a logarithmic dependence[28,38]. The resource-replete laboratory experiments likely give an upper limit of the significance of active uptake, because high light intensity leads to high rates of active uptake[38] and laboratory experiments typically feature much higher light intensities than those that characterise the deep chlorophyll maximum at which Noelaerhabdaceae density is highest in the oligotrophic open ocean ocean[39]. In fact, this more limited influence of active uptake in the deep oligotrophic ocean has been identified in measurements of ε_p on alkenones through depth profiles in the water column[40]. A first simulation employs a logarithmic dependence of chloroplast HCO_3^- transport on [CO_{2aq}], with a slope similar to that observed in cultures but an intercept half that observed in cultures to account for lower light intensity (Fig. 8). A second simulation supplements HCO_3^- supply to the chloroplast in terms of reallocation of HCO_3^- from calcification to photosynthesis. The HCO_3^- available for reallocation to the chloroplast is estimated as the difference between the HCO_3^- flux to the coccolith vesicle which

would be required to match $\varepsilon_{coccolith}$ if coccolith calcification remained constant, from that required to match $\varepsilon_{coccolith}$ for the observed situation of reduced calcification per cell surface area in the last 8 Ma (reduced PIC/POC) for the coccoliths less than 5 μm. Both simulations parameterize HCO_3^- uptake across the cytosol as 0.5x the sum of HCO_3^- transport to the coccolith vesicle and chloroplast, analogous to previously published simulations[28]. Large cells also show evidence of shifts in HCO_3^- allocation to calcification: a major decrease in $\varepsilon_{coccolith}$, an indicator of coccolith vesicle HCO_3^- influx relative to calcification, occurs after 8 Ma (Fig. 5). This reallocation in large coccolithophores probably had a minimal effect on the alkenone ε_p record, because by 8 Ma mean Noëlaerhabdaceae size has already decreased significantly, implying that the majority of alkenones were produced by small cells rather than large cells. However, in both large and small cells, the 10-6 Ma onset of this potential HCO_3^- reallocation to the chloroplast is similar to the onset inferred for increased HCO_3^- to the chloroplast from a simple dependence of active uptake on CO_2 concentrations described as simulation 1, so would be unlikely to modify the simulations of active uptake.

Calculating constraints on past ocean pH and alkalinity

We assess the constraints on the magnitude of potential surface alkalinity increase which are provided by pH estimates from ref. 3 for the last 2 Ma and pCO_2 values from Vostok ice cores for the last 800 ka[4,41,42] (Supplementary Fig. 6). We use pH determinations made at ODP Site 668 in the eastern equatorial Atlantic, a region with CO_2 in equilibrium with the atmosphere[3]. Over the last 800 ka, atmospheric CO_2 during glacial maxima consistently declines to 180 ± 10 ppmv[4], coherent with glacial temperature anomalies in Antarctica. If this 180 ppmv level of pCO_2 were characteristic of glacial maxima during the preceding 1200 kyr, then pH and CO_2 could be used to estimate surface alkalinity at the location of ODP 688 during glacial times. Calculations were performed with the programme CO2sys[43], using constants of ref. 44. Under these assumptions, surface alkalinity at this site could have increased substantially during glacials since the mid-Pleistocene, from 1800 to 2300 μmol kg^{-1}, an increase of nearly 30 % over the last 0.6 Ma (Supplementary Fig. 6c). Among interglacials of the past 800 ka, pCO_2 has varied much more significantly from 240 to 300 ppmv, with higher interglacial pCO_2 in the last 500 ka. Consequently, one cannot make an analogous assumption of uniform interglacial CO_2 prior to the ice core record to estimate long-term interglacial alkalinity changes prior to 800 ka. During the period of direct pCO_2 determinations in ice cores, calculated alkalinity during interglacials is lower than that during glacial periods.

As estimates of surface water pH from B isotopic ratios of monospecific foraminiferal samples are available only discontinuously over the last 15 Ma, as shown in Figure 7f, we employ the more complete records of variation in ocean carbon chemistry are available for $[CO_3^{-2}]$ to estimate long term alkalinity change. Long term variations in surface ocean $[CO_3^{-2}]$ have been derived from coupled records of seawater calcium concentration and the history of the carbonate compensation depth (CCD)[7]. These estimates are subject to some uncertainty due to the potential decoupling of the CCD and the carbonate saturation horizon which is the parameter strictly regulated by $[CO_3^{-2}]$[45]. Overall this record suggests increasing $[CO_3^{-2}]$ over the last 15 Ma (Supplementary Fig. 7). The combination of $[CO_{2aq}]$ records with these $[CO_3^{-2}]$ reconstructions suggests a relatively stable total alkalinity from about 15 to 2 Ma and a progressively increasing surface ocean pH (Supplementary Fig. 7). For discrete intervals between 15 to 12 Ma, available direct pH estimates from planktic

foraminifera $\delta^{11}B$ suggest the potential for more dynamic carbon system variations over timescales of a few million years. Therefore, within the uncertainties of existing data between 15 and 2 Ma, we cannot rule out the possibility of variations in alkalinity that may have contributed to changes in SN coccolith thickness during this interval.

Supplementary References

1 Young, J. & Ziveri, P. Calculation of coccolith volume and its use in calibration of carbonate flux estimates. *Deep Sea Research Part II* **47**, 1679–1700 (2000).

2 Henderiks, J. Coccolithophore size rules - Reconstructing ancient cell geometry and cellular calcite quota from fossil coccoliths. *Marine Micropaleontology* **67**, 143-154 (2008).

3 Hönisch, B., Hemming, N. G., Archer, D., Siddall, M. & McManus, J. F. Atmospheric carbon dioxide concentration across the mid-Pleistocene transition. *Science* **324**, 1551-1554 (2009).

4 Lüthi, D. *et al*. High-resolution carbon dioxide concentration record 650,000–800,000 years before present. *Nature* **453** 379-382 (2008).

5 Chen, M.-T. Late Quaternary paleoceanography of the equatorial Indo-Pacific Ocean: A quantitative analysis based on marine micropaleontological data; *PhD thesis*, Brown Univeristy (1994).

6 Curry, W. B. & Oppo, D. W. Synchronous, high-frequency oscillations in tropical sea surface temperatures and North Atlantic Deep Water productivity during the last glacial cycle. *Paleoceanography* **12**, 1-14 (1997).

7 Tyrrell, T. & Zeebe, R. E. History of carbonate ion concentration over the last 100 million years. *Geochimica et Cosmochimica Acta* **68**, 3521-3530 (2004).

8 Seki, O. *et al*. Alkenone and boron-based Pliocene pCO_2 records. *Earth and Planetary Science Letters* **292**, 201–211 (2010).

9 Hönisch, B., Hemming, N. G., Archer, D., Siddall, M. & McManus, J. F. Atmospheric carbon dioxide concentration across the mid-Pleistocene transition. *Science* **324**, 1551-1554 (2009).

10 Foster, G. L., Lear, C. H. & Rae, J. W. B. The evolution of pCO_2, ice volume and climate during the Middle Miocene. *Earth and Planetary Science Letters* **341-344**, 243-254 (2012).

11 Smith, H. E. K. *et al*. Predominance of heavily calcified coccolithophores at low $CaCO_3$ saturation during winter in the Bay of Biscay. *Proceedings of the National Academy of Sciences USA* **109**, 8845-8849 (2012).

12 Bach, L. T. *et al*. Dissecting the impact of CO_2 and pH on the mechanisms of photosynthesis and calcification in the coccolithophore *Emiliania huxleyi*. *New Phytologist* **199**, 121-134, doi:doi: 10.1111/nph.12225 (2013).

13 Popp, B. N. *et al*. Effect of phytoplankton cell geometry on carbon isotopic fractionation. *Geochimica y Cosmochimica Acta* **62**, 69-77 (1998).

14 Langer, G. *et al*. Species-specific responses of calcifying algae to changing seawater carbonate chemistry. *Geochemistry Geophysics Geosystems* **7** (2006).

15 Langer, G., Nehrke, G., Probert, I., Ly, J. & Ziveri, P. Strain-specific responses of *Emiliania huxleyi* to changing seawater carbonate chemistry. *Biogeosciences* **6**, 2637-2646, doi:10.5194/bg-6-2637-2009 (2009).

16 Rost, B., Zondervan, I. & Riebesell, U. Light-dependent carbon isotope fractionation in the coccolithophorid *Emiliania huxleyi*. *Limnology and Oceanography* **47**, 120-128 (2002).

17 De Bodt, C., Van Oostende, N., Harlay, J., Sabbe, K. & Chou, L. Individual and interacting effects of pCO_2 and temperature on *Emiliania huxleyi* calcification: study of the calcite

production, the coccolith morphology and the coccosphere size. *Biogeosciences* **7**, 1401-1412 (2010).
18 Iglesias-Rodriguez, M. D. *et al*. Phytoplankton calcification in a high-CO_2 world. *Science* **320**, 336-340 (2008).
19 Langer, G. & Bode, M. CO_2 mediation of adverse effects of seawater acidification in *Calcidiscus leptoporus*. *Geochemistry, Geophysics, Geosystems* **12** (2011).
20 Rickaby, R. E. M., Henderiks, J. & Young, J. N. Perturbing phytoplankton: response and isotopic fractionation with changing carbonate chemistry in two coccolithophore species. *Climate of the Past* **6**, 771–785 (2010).
21 Müller, M. N. *et al*. Influence of CO_2 and nitrogen limitation on the coccolith volume of *Emiliania huxleyi* (Haptophyta). *Biogeosciences* **9**, 4155-4167 (2012).
22 Fuertes, M. A., Flores, J. A. & Sierro, F. J. The use of circularly polarized light for biometry, identification and estimation of mass of coccoliths. *Marine Micropaleontology* **113**, 44-55 (2014).
23 Bollmann, J. Technical Note: Weight approximation of coccoliths using a circular polarizer and interference colour derived retardation estimates (The CPR Method). *Biogeosciences* **11**, 1899-1910, doi:10.5194/bg-11-1899-2014 (2014).
24 Beaufort, L., Barbarin, N. & Gally, Y. Optical measurements to determine the thickness of calcite crystals and the mass of thin carbonate particles such as coccoliths. *Nature Protocols* **9**, 633-642 (2014).
25 Bickert, T., Haug, G. & Tiedemann, R. Late Neogene benthic stable isotope record of ODP Site 999: Implications for Caribbean paleoceanography, organic carbon burial and the Messinian Salinity Crisis. *Paleoceanography* **19**, PA1023 (2004).
26 Haug, G. H. & Tiedemann, R. Effect of the formation of the Isthmus of Panama on Atlantic Ocean thermohaline circulation. *Nature* **393** (1998).
27 Zachos, J. C., Dickens, G. R. & Zeebe, R. E. An early Cenozoic perspective on greenhouse warming and carbon-cycle dynamics. *Nature* **451**, 279-283 (2008).
28 Bolton, C. T. & Stoll, H. M. Late Miocene threshold response of marine algae to carbon dioxide limitation. *Nature* **500**, 558-562 (2013).
29 Hopkinson, B. M., Dupont, C. L., Allen, A. E. & Morel, F. M. M. Efficiency of the CO_2-concentrating mechanism of diatoms. *Proceedings of the National Academy of Sciences USA* **108**, 3830-3837 (2011).
30 Kameo, K. & Bralower, T. J. in *Proc. ODP, Sci. Results, 165* (eds R.M. Leckie, H. Sigurdsson, G.D. Acton, & G. Draper) 3-17, Ocean Drilling Program (2000).
31 Young, J. R. Size variation of Neogene *Reticulofenestra* coccoliths from Indian Ocean DSDP cores. *Journal of Micropalaeontology* **9**, 71-85 (1990).
32 Henderiks, J. & Pagani, M. Refining ancient carbon dioxide estimates: Significance of coccolithophore cell size for alkenone-based pCO_2 records. *Paleoceanography* **22** (2007).
33 Henderiks, J. & Pagani, M. Coccolithophore cell size and the Paleogene decline in atmospheric CO_2. *Earth and Planetary Science Letters* **269**, 576-584 (2008).
34 Aloisi, G. Co-variation of metabolic rates and cell-size in coccolithophores. *Biogeosciences Discussions* **12**, 6215-6284 (2015).
35 Stoll, H. M. & Schrag, D. P. Coccolith Sr/Ca as a new indicator of coccolithophorid calcification and growth rate. *Geochemistry Geophysics Geosystems* **1**, 1-24 (2000).
36 Bolton, C. T. *et al*. Glacial–interglacial productivity changes recorded by alkenones and microfossils in late Pliocene eastern equatorial Pacific and Atlantic upwelling zones. *Earth and Planetary Science Letters* **295**, 401-411 (2010).
37 Sosdian, S. M. *et al*. Cenozoic seawater Sr/Ca evolution. *Geochemistry Geophysics Geosystems* **13**, Q10014, doi:doi:10.1029/2012GC004240 (2012).

38 Cassar, N., Laws, E. A. & Popp, B. N. Carbon isotopic fractionation by the marine diatom *Phaeodactylum tricornutum* under nutrient- and light-limited growth conditions. *Geochimica et Cosmochimica Acta* **70**, 5323-5335 (2006).
39 Beaufort, L., Couapel, M., Buchet, N., Claustre, H. & Goyet, C. Calcite production by coccolithophores in the south east Pacific Ocean. *Biogeosciences* **5**, 1101-1117 (2008).
40 Tolosa, I. *et al*. Distribution of lipid biomarkers and carbon isotope fractionation in contrasting trophic environments of the South East Pacific. *Biogeosciences* **5**, 949-968 (2008).
41 Petit, J.-R. *et al*. Climate and atmospheric history of the past 420,000 years from the Vostok ice core, Antarctica. *Nature* **399**, 429-436 (1999).
42 Siegenthaler, U. *et al*. Stable carbon cycle–climate relationship during the late Pleistocene. *Science* **310**, 1313-1317 (2005).
43 Program developed for CO_2 systems calculations (Oak Ridge, TN, USA: ORNL/CDIAC-105 Carbon Dioxide Information Analysis Centre, Oak Ridge National Laboratory, US Department of Energy, 1998).
44 Roy, R. *et al*. The dissociation constants of carbonic acid in seawater at salinities 5 to 45 and temperatures 0 to 45°C. *Marine Chemistry* **44**, 249-267 (1993).
45 Greene, S. *et al*. Long-term stability of the carbonate compensation depth across the Late Paleocene-Early Eocene warming trend, presented at *Goldschmidt* (2014).
